# Giant cell tumor of bone inhibits osteoblastogenesis via WNT5B

**DOI:** 10.1007/s00774-026-01713-3

**Published:** 2026-03-23

**Authors:** Masaki Shimada, Tomonori Tsuyama, Naoto Yoshimura, Makoto Tateyama, Hideto Matsunaga, Fuka Homma, Kasumi Dainobu, Xiao Tian, Shu Takata, Kosei Takata, Shuntaro Tanimura, Yuto Shibata, Kazuya Maeda, Junki Kawakami, Takahiro Arima, Tatsuki Karasugi, Takuya Tokunaga, Hiro Sato, Tetsuro Masuda, Satoshi Hisanaga, Yuki Kai, Soichiro Karata, Hikaru Goshogawa, Rui Tajiri, Hibiki Yamada, Yusuke Uehara, Takayuki Nakamura, Masaki Yugami, Kazuki Sugimoto, Ryuji Yonemitsu, Hironori Tanoue, Kazuya Yamagata, Takeshi Miyamoto

**Affiliations:** 1 Department of Orthopedic Surgery, Faculty of Life Sciences, Kumamoto University, 1-1-1 Honjo, Chuo-Ku, Kumamoto, 860-8556 Japan; 2 Center for Metabolic Regulation of Healthy Aging (CMHA), Faculty of Life Sciences, Kumamoto University, 1-1-1 Honjo, Chuo-Ku, Kumamoto, 860-8556 Japan; 3 Department of Dentistry and Oral Surgery, Keio University, School of Medicine, 35 Shinano-Cho, Shinjuku-Ku, Tokyo, 160-8582 Japan; 4 Department of Oral and Maxillofacial Surgery, Kumamoto University, 1-1-1 Honjo, Chuo-Ku, Kumamoto, 860-8556 Japan; 5 Department of Medical Biochemistry, Faculty of Life Sciences, Kumamoto University, 1-1-1 Honjo, Chuo-Ku, Kumamoto, 860-8556 Japan

**Keywords:** Giant cell tumor of bone, WNT5B, Mineralization

## Abstract

**Introduction:**

Giant cell tumor of bone (GCTB) induces overproduction of bone-resorbing osteoclasts through receptor activator of nuclear factor kappa B ligand (RANKL), leading to bone resorption and destruction. Consequently, denosumab, a neutralizing antibody against RANKL (a cytokine essential for osteoclast induction), is used to treat patients with GCTB. However, the activity of bone formation in GCTB remains poorly understood. Here, we show that GCTB antagonizes bone formation by expressing WNT5B, which inhibits bone formation.

**Materials and methods:**

Co-culture of NCC-GCTB1-C1 (GCTB1s), a human GCTB cell line, with human adipose-derived stem cells (ADSCs) was performed with osteoblast induction medium. To identify the inhibitors of osteoblast differentiation, we reanalyzed the single-cell RNA sequencing data that was previously published. In addition, we performed spatial transcriptome analysis (Visium) against the section of paraffin block of GCTB. The targeted protein was knocked out using CRISPR/Cas9 and co-culture was performed.

**Results:**

Co-culture of GCTB1s with ADSCs significantly inhibited mineralization of ADSCs. Reanalysis of single-cell RNA sequencing data indicated that GCTB tumors express *WNT5B,* and we observed that GCTB1s express *WNT5B*. We then knocked out *WNT5B* in GCTB1s using CRISPR/Cas9 and co-cultured them with ADSCs and observed significant rescue of mineralization in ADSCs relative to ADSCs cultured with GCTB1s expressing *WNT5B*. We also show that ADSC supernatants induce mineralization of GCTB1s.

**Conclusion:**

These studies indicate that GCTB not only induces osteoclasts, but also possesses activity that inhibits bone formation.

**Supplementary Information:**

The online version contains supplementary material available at 10.1007/s00774-026-01713-3.

## Introduction

Giant cell tumor of bone (GCTB) induces giant osteoclast cells in bone and is diagnosed by histological identification of those cells [1, 2]. While osteoclasts are of the monocyte–macrophage lineage and derived from hematopoietic stem cells, GCTB is thought to be derived from mesenchymal stem cells that express receptor activator of nuclear factor kappa B ligand (RANKL), a cytokine essential to induce osteoclast differentiation [3]. In fact, recently, the histone mutation H3.3 G34W has been identified in GCTB, and anti-H3.3 G34W monoclonal antibodies reportedly stain surrounding stromal cells, not the giant cells [4].

GCTB typically occurs in relatively young individuals aged 20–40 years and develops in various bones, including those near joints [5]. Due to its high recurrence rate and potential for distant metastasis, it is classified as an intermediate malignant tumor [6]. Surgical resection is the standard GCTB treatment, but recurrence frequently happens, and tumors may occur in bones where resection is difficult [5, 7]. Consequently, there is an urgent need to develop drug therapies for GCTB. GCTB induces significant bone resorption, bone destruction, and pain via osteoclast formation [8]. Therefore, denosumab, a neutralizing antibody against RANKL, is sometimes administered to inhibit bone resorption in patients with GCTB [9]; however it is not a curative for the tumor itself, but rather suppresses osteoclast formation induced by GCTB. Therefore, identifying potential new therapeutic targets for GCTB remains a challenge.

Bone homeostasis is maintained by a delicate balance between the activity of osteoclasts, which resorb bone, and osteoblasts, which form bone [10]. The transcription factor Runx2 is essential for osteoblast differentiation: Runx2-deficient mice exhibit complete inhibition of bone formation [11]. Osteoblasts express RANKL and activate bone remodeling [12]. Osteoclasts activate or release growth factors such as TGFβ and IGF-1 stored in bone extracellular matrix proteins during bone resorption, activating osteoblast-mediated bone formation [13]. Thus, activation of bone resorption also activates bone formation. In bone loss diseases such as osteoporosis, induced by RANKL, the number of osteoclasts increases, leading to heightened osteoblast activity [14]. However, in those conditions, osteoclast activity exceeds osteoblast activity, promoting a lag in bone formation and decreased bone mass [15].

Among factors known to inhibit bone formation, some of Wnt, Frizzled, and DKK-related factors are recognized as particularly potent bone formation inhibitors [16–18]. Another factor is sclerostin, and romosozumab, a monoclonal neutralizing antibody against sclerostin, is used to treat osteoporosis [19, 20]. The Wnt/β-catenin pathway is also known to activate bone formation [18]. β-Catenin was reportedly detected in the nuclei of both stromal mononuclear cells (SCs) and osteoclast-like giant cells (GCs) within the GCTB tissue [21]. Histological studies in GCTB patients treated with denosumab demonstrated that the Wnt/β-catenin pathway was activated in stromal cells during tissue ossification induction [22]. Furthermore, GCTB tumor stromal cells derived from GCTB patients exhibited osteoblast-like characteristics, and β-catenin activation was demonstrated to be required for their osteoblast differentiation in vitro [22]. These findings suggest that the Wnt/β-catenin pathway is activated in GCTB tissue and may induce osteoblast differentiation and ossification. However, the specific Wnt molecules expressed in GCTB tissue or the inhibitory signaling pathways for osteoblast differentiation in GCTB were not well characterized.

In GCTB, excessive osteoclast induction due to tumor-produced RANKL is thought to cause excessive bone resorption and bone destruction [23]. However, little is known about how GCTB impacts bone formation.

Single-cell RNA sequencing enables comprehensive detection of transcripts expressed in heterogeneous cell populations and allows clustering of cells based on transcript expression profiles. Recently, efforts have also emerged to identify novel functional factors by reanalyzing previously published single-cell RNA sequencing data [24–26]. Furthermore, data analysis using Visium, a spatial transcriptomics platform, enables comprehensive spatial transcriptome assessment of tissue specimens [27]. Combining these approaches has made it possible to characterize specific cell populations and define their localization within tissues. This study employs these techniques to elucidate the effects of GCTB on the osteogenic system, and identified WNT5B, which is an inhibitor of osteoblastogenesis, as highly expressed by GCTB cells.

## Materials and methods

### Reagents

The primary antibodies anti-histone H3.3 G34W rabbit monoclonal antibody (31–1145-00-S, RevMAb Biosciences, Burlingame, CA, USA), anti-WNT5A/B rabbit polyclonal antibody (55184–1-AP, Proteintech, Rosemont, IL, USA), and anti-non-phospho (active) β-catenin (Ser33/37/Thr41) rabbit monoclonal antibody (8814T, Cell Signaling Technology, Danvers, MA, USA) were purchased from the indicated companies. Recombinant human WNT5B (rWNT5B) protein (7347-WN-025) was purchased from R&D Systems (Minneapolis, MN, USA).

### Cells and culture

The GCTB cell lines NCC-GCTB1-C1 (RRID: CVCL_A9QC) and NCC-GCTB3-C1 (RRID: CVCL_A0SS), described here as GCTB1s and GCTB3s, respectively, were purchased from Bizcom (Tokyo, Japan). Human adipose-derived stem cells (PT-5006, Basel, Switzerland) were purchased from Lonza. Cells were cultivated in DMEM (08459–35, Nacalai Tesque, Kyoto, Japan) supplemented with 10% FBS (Biowest, Nuaillé, France) and 1% penicillin–streptomycin (26253-84, Nacalai Tesque, Kyoto, Japan).

### Osteoblast differentiation assay

Cells were cultured in osteogenic induction medium containing 10% FBS, 1% penicillin–streptomycin, 1% ascorbic acid, 2% β-glycerophosphate, and 0.2% hydrocortisone. The medium was changed every 2–3 days, and differentiation was confirmed by Alizarin Red S staining.

ADSCs were plated into 96-well plates at the density of 5 × 10^3^ cells/well and cultivated in osteogenic induction medium with or without rWNT5B protein for 21 days. The medium was changed every 2–3 days, and differentiation was confirmed by Alizarin Red S staining.

### Co-culture assay

NCC-GCTB1-C1 (GCTB1s) and human adipose-derived stem cells (ADSCs) were co-cultured in horizontal co-culture plates (NICO-1, Ginrei Lab, Ishikawa, Japan), a system that allows passage of small molecules or secreted factors through polycarbonate membranes with 1.2 µm pores (380–19261, Ginrei Lab, Ishikawa, Japan), but blocks movement of cells between chambers. Osteogenic induction medium was used as described above.

### Collection of ADSC supernatants

ADSCs were seeded into 24-well plates at a density of 2 × 10^4^ cells/well and cultivated in osteogenic induction medium until they underwent differentiation. Culture supernatants were collected on days 0, 7, 21, and 24, centrifuged for 5 min at 273×*g,* and stored at −20℃ until further use.

### RNA isolation and qPCR

Total RNA was isolated by TRI Reagent (TR118, Molecular Research Center, Cincinnati, OH, USA), and cDNA was obtained using PrimeScript RT Master Mix (RR036A, Takara, Shiga, Japan) according to the manufacturer’s instructions. qPCR was performed using TB Green Premix Ex Taq II (Tli RNaseH Plus) (RR820A, Takara) on the Thermal Cycler Dice Real Time System III (TP950, Takara). Cycling conditions were initiated at 95°C for 30 s, followed by 40 cycles of 95°C for 30 s and 60°C for 30 s. Relative expression of target transcripts was normalized to *ACTB* mRNA expression. Primer sequences are listed as below.

*ACTB* forward: 5′- GCCCTGAGGCACTCTTCCA -3′.

*ACTB* reverse: 5′- CGGATGTCCACGTCACACTTC -3′.

*RUNX2* forward: 5′-CGGAATGCCTCTGCTGTTAT-3′.

*RUNX2* reverse: 5′-AGCTTCTGTCTGTGCCTTCT-3′.

*ALPL* forward: 5′-CCACGTCTTCACATTTGGTG-3′.

*ALPL* reverse: 5′-AGACTGCGCCTGGTAGTTGT-3′.

*COL1A1* forward: 5′-GCCTCCCAGAACATCACCTA-3′.

*COL1A1* reverse: 5′-TGGGATGGAGGGAGTTTACA-3′.

*BGLAP* forward: 5′-AGGTGCAGCCTTTGTGTCCA-3′.

*BGLAP* reverse: 5′-GGCTCCCAGCCATTGATACAG-3′.

*WNT5A* forward: 5′-ATTCTGGCTCCACTTGTTGCT-3′.

*WNT5A* reverse: 5′-GTTATTCATACCTAGCGACCACCA-3′.

*WNT5B* forward: 5′-GGGAGCGAGAGAAGAACTTTGC-3′.

*WNT5B* reverse: 5′-GCAGGCTACGTCTGCCATCTTA-3′.

*SFRP4* forward: 5′-GAGTGGCGCTCAAGGATGAT-3′.

*SFRP4* reverse: 5′-TGTCCTGAACTGTTCTCCGC-3′.

*CTNNB1* forward: 5′-ATGGAACCAGACAGAAAAGC-3′.

*CTNNB1* reverse: 5′-GCTACTTGTTCTTGAGTGAAG-3′.

### Immunohistochemical staining

Paraffin-embedded blocks of GCTB tissue resected at Kumamoto University Hospital were used for immunohistochemical staining. Sections were deparaffinized and antigen retrieval was performed in 95°C citrate buffer for 40 min. Subsequently, internal peroxidase activities were blocked with 3% hydrogen peroxide for 15 min and nonspecific antigens were blocked with 5% normal goat serum and 0.3% Triton X-100 in PBS for 30 min at room temperature. Then, primary antibodies, namely, anti-H3.3 G34W (1: 500) or anti-WNT5A/B (1: 100) antibodies, were applied to sections and incubated at 4°C overnight. The secondary antibody, HRP-conjugated goat anti-rabbit polyclonal immunoglobulins (P0448, Agilent, Santa Clara, CA, USA), was then applied and DAB (MK210, Takara, Shiga, Japan) staining was performed with hematoxylin counterstaining.

### Immunofluorescence staining

GCTB1s were cultured on chamber slides. Five minutes after adding supernatants collected from ADSCs on day 24 post-differentiation or standard osteogenic induction (control) medium, cultured cells were fixed for 10 min with 4% paraformaldehyde. As a control for activation of Wnt/β-catenin signaling, LiCl was added to standard osteogenic induction medium at a concentration of 50 mM for 5 min.

After blocking nonspecific antigens as described above, anti-non-phospho (active) β-catenin antibody diluted 1:1000 was applied and incubated at 4°C overnight. Secondary goat anti-rabbit IgG (Alexa Fluor 488) (ab150077, Abcam, Cambridge, UK) was then added, followed by DAPI (ib50011, Nippon Genetics, Tokyo, Japan) counterstaining.

### Western blot analysis

ADSCs or GCTB1s were cultured with DMEM containing penicillin–streptomycin without FBS in 24-well culture plates. After 24 h, each supernatant was collected and centrifuged at 273×*g* for 5 min, and the resulting supernatants were collected for further use. Proteins were extracted from supernatants using chloroform/methanol [28] and dissolved in 1% SDS and sample buffer solution with 3-mercapto-1,2-propanediol (× 2) (199–16132, Wako, Osaka, Japan). Total protein was subjected to SDS-PAGE (12%) and blotted onto polyvinylidene difluoride (PVDF) membranes (1620175, Bio-Rad, Hercules, CA, USA). Coomassie Brilliant Blue (CBB) staining served as a loading control. Membranes were incubated with primary anti-WNT5A/B antibody diluted 1:1000, followed by secondary HRP-conjugated goat anti-rabbit polyclonal immunoglobulins (P0448, Agilent, Santa Clara, CA, USA). Reactive bands were visualized using Amersham ECL Select Western Blotting Detection Reagent (RPN2235, Cytiva, Uppsala, Sweden).

### Gene knockout (KO) in GCTB1s using CRISPR/Cas9

WNT5B KO GCTB1s were generated using a lentiviral CRISPR/Cas9 system. The lentiviral pLV[2CRISPR]-hCas9:T2A:Puro-U6 expression vector (VectorBuilder, Chicago, IL, USA) containing either the sequence of human *WNT5B* gRNA or scramble gRNA was used. *WNT5B* gRNA sequences were as follows:

GGTCCTTGGGCCGCGCCGTC,

GTACGGCTACCGCTTCGCCA.

### Alizarin Red S staining to detect mineralization

After differentiation, cultured cells were fixed with 4% paraformaldehyde for 10 min, followed by 15 min incubation with 1% Alizarin Red S (pH 6.3). After washing with distilled water, the stain was eluted in 5% formic acid and the absorbance at 450 nm was measured.

### Processing of scRNA-seq data

To assess the characteristics of stromal cells in GCTB, raw data were downloaded from GSE149211 (Khazaei et al. 2020) in the Gene Expression Omnibus (GEO) database. The data included four raw FASTQ files of GCTB species: GSM4492128, GSM4492129, GSM4492130, and GSM4492131. Each file was reanalyzed. Raw reads were aligned to the GRCh38 reference genome, and unique molecular identifier (UMI) counting was performed using Cell Ranger Package (version 7.0.1) pipeline with default parameters. The Seurat package (version 5.0.1) was used for quality filtering and subsequent analysis. Low-quality cells (> 5,500 genes/cell and 10% mitochondrial genes) were excluded. Subsequently, 12,947 cells (GSM4492128), 1,730 cells (GSM4492129), 11,327 cells (GSM4492130), and 7,627 cells (GSM4492131) remained. To integrate all four samples, we also used Seurat’s “FindIntegrationAnchors” and “IntegrateData” functions. Next, principal component analysis (PCA) with the “RunPCA” function was performed to reduce dimensionality of scRNA-seq data. To identify clusters, the top 20 principal components were selected and subjected to “FindNeighbors”, “FindClusters”, and “RunUMAP” (Uniform Manifold Approximation and Projection [UMAP]) functions. The resolution parameter was set to 0.5 unless otherwise specified. Cluster-specific marker genes were identified using the Wilcoxon test implemented in “FindAllMarkers” function. Cell clusters were manually annotated based on marker gene analysis.

### Visium spatial transcriptomic analysis

To perform Visium CytAssist Spatial Gene Expression analysis (10 × Genomics), a 5 µm section of formalin-fixed paraffin-embedded (FFPE) tumor blocks was placed on a standard glass slide and deparaffinized, H&E-stained, and imaged according to the manufacturers’ protocols. After decross-linking, probe hybridization and ligation were performed. Using Visium CytAssist, ligated probes were released and captured on a 6.5 mm × 6.5 mm capture area of the Visium slide. Captured probes were extended and carried for library preparation. The constructed libraries were sequenced by NovaSeq 6000 (Illumina). The Space Ranger pipeline (version 2.0.0, 10 × Genomics) was used to process Visium spatial gene expression data, including the generation of FASTQ files, alignment to the GRCh38 human reference genome, and barcode/UMI counting. Further processing and visualization were performed with the Seurat package (version 5.0.1). The tissue slice covered 4,992 unique spots with a median of 6,623 genes detected. Low-quality spots (> 20% mitochondrial genes) were excluded. The “SCTransform” function in Seurat was used for normalization, and PCA with “RunPCA” was used for dimensionality reduction. To identify clusters, the top 30 principal components were selected and subjected to “FindNeighbors”, “FindClusters”, and “RunUMAP” functions.

### Statistical analysis

Normal data distribution was confirmed before performing analysis with Welch’s *t*-test (2 groups) or the Games–Howell test (3 or more groups). A Mann–Whitney *U* test was used when normal distribution was not assumed. A *p* value < 0.05 was considered statistically significant.

### Ethics and consent

This study was approved by the ethics committee at the Faculty of Life Sciences of Kumamoto University and consents were obtained using the opt-out method. We obtained paraffin blocks from the Department of Pathology at our hospital on July 25, 2022, July 10, 2023, and January 22, 2025. During the period in which the paraffin blocks were borrowed, access to individual patient data was possible; however, we did not access any personally identifiable information.

## Results

### A soluble factor expressed by GCTB1s significantly inhibits ADSC mineralization

**Fig. 1:**
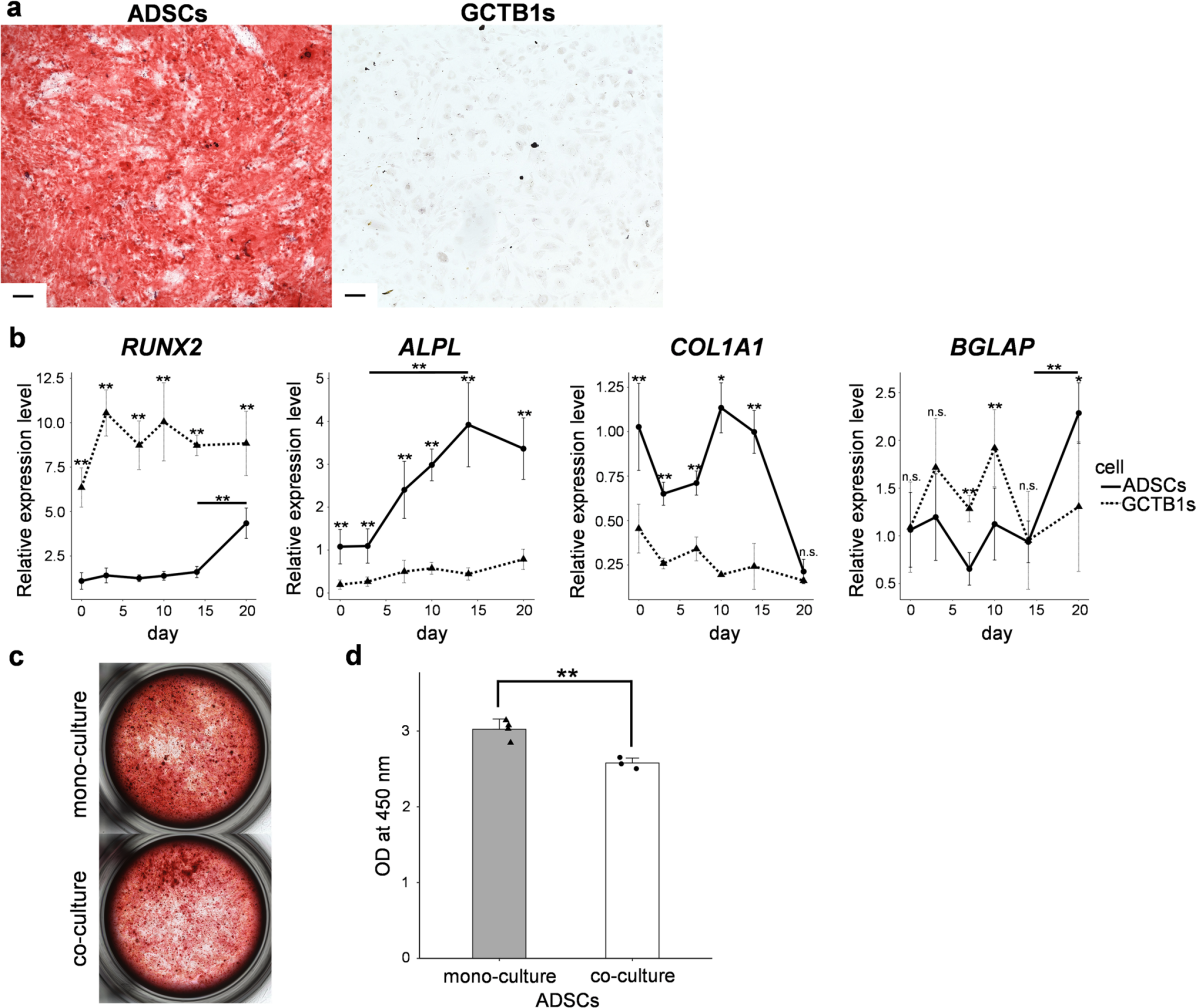
Soluble factors secreted by GCTB1s significantly inhibit ADSC mineralization. **a** and **b** ADSCs and GCTB1s were cultured separately in osteogenic induction medium and then **a** stained with Alizarin Red S on day 22 (bar = 200 µm) and **b** analyzed by qPCR on days 3, 7, 10, 14, or 20. For qPCR, shown is the mean indicated gene expression relative to *β-actin* (*ACTB*) ± SD in each cell (n = 6 each, Mann–Whitney *U* test: **p < 0.01, *p < 0.05). **c** and **d** ADSCs and GCTB1s were cultured with osteogenic induction medium in a horizontal co-culture system for 20 days. ADSCs were then stained with Alizarin Red S to assess mineralization as an indicator of differentiation (**c**). ADSC mono-culture in the presence of osteogenic induction medium served as a control. To quantify mineralization, Alizarin Red S was extracted from all samples in 5% formic acid and assessed for absorbance at 450 nm (**d**). Data represent mean absorbance ± SD (mono-culture, n = 4; co-culture, n = 3, Welch’s *t*-test: **p < 0.01)

To analyze the potency for osteoblastic differentiation, we cultured NCC-GCTB1-C1 (GCTB1s) or human adipose-derived stem cells (ADSCs) in osteogenic induction medium and evaluated mineralization by Alizarin Red S staining on day 22. Based on staining analysis, treatment with osteogenic induction medium induced mineralization in ADSCs, but not in GCTB1s (Fig. 1a). To assess expression of osteoblastic genes during osteoblastic differentiation induction in both GCTB1s and ADSCs, we cultured each cell type in osteogenic induction medium and after 20 days harvested mRNA for qPCR analysis (Fig. 1b). In ADSCs, *ALPL* mRNA expression increased significantly with osteoblastic differentiation (Fig. 1b). At later stages of differentiation, *RUNX2* and *BGLAP* transcript levels increased in ADSCs, confirming that osteoblastic differentiation occurred in late culture phases (Fig. 1b). In contrast, GCTB1s exhibited significantly lower expression of *COL1A1* and *ALPL* mRNAs compared to ADSCs and did not show increased *BGLAP* transcript levels at late differentiation stages (Fig. 1b). Interestingly, *RUNX2* mRNA was highly expressed in GCTB1s throughout the culture period, consistent with previous reports [29, 30]. We observed similar results in GCTB3s, a different GCTB cell line (Supplementary Fig. 1).

Next, we cultured GCTB1s and ADSCs separately in standard medium for 2 days and then switched them to osteogenic induction medium. On day 7 after the switch, we connected culture wells for both cell types using a 1.2 µm pore size filter to establish a horizontal co-culture system, allowing continuous medium flow through the filter but preventing passage of cells. Seven days after establishing this system, we began culturing cells separately in osteogenic induction medium and, on day 20 after the switch to osteogenic induction medium, performed Alizarin Red S staining to assess mineralization (Fig. 1c). Control ADSCs were cultured in a similar horizontal co-culture system, but the adjoining well was cell free to simulate mono-culture. ADSCs co-cultured horizontally with GCTB1s showed significantly suppressed mineralization relative to controls at culture day 20 (Fig. 1d), suggesting that GCTB1s express soluble factors that inhibit mineralization of ADSCs.

### GCTB express WNT5B, an inhibitor of osteoblast differentiation

**Fig. 2:**
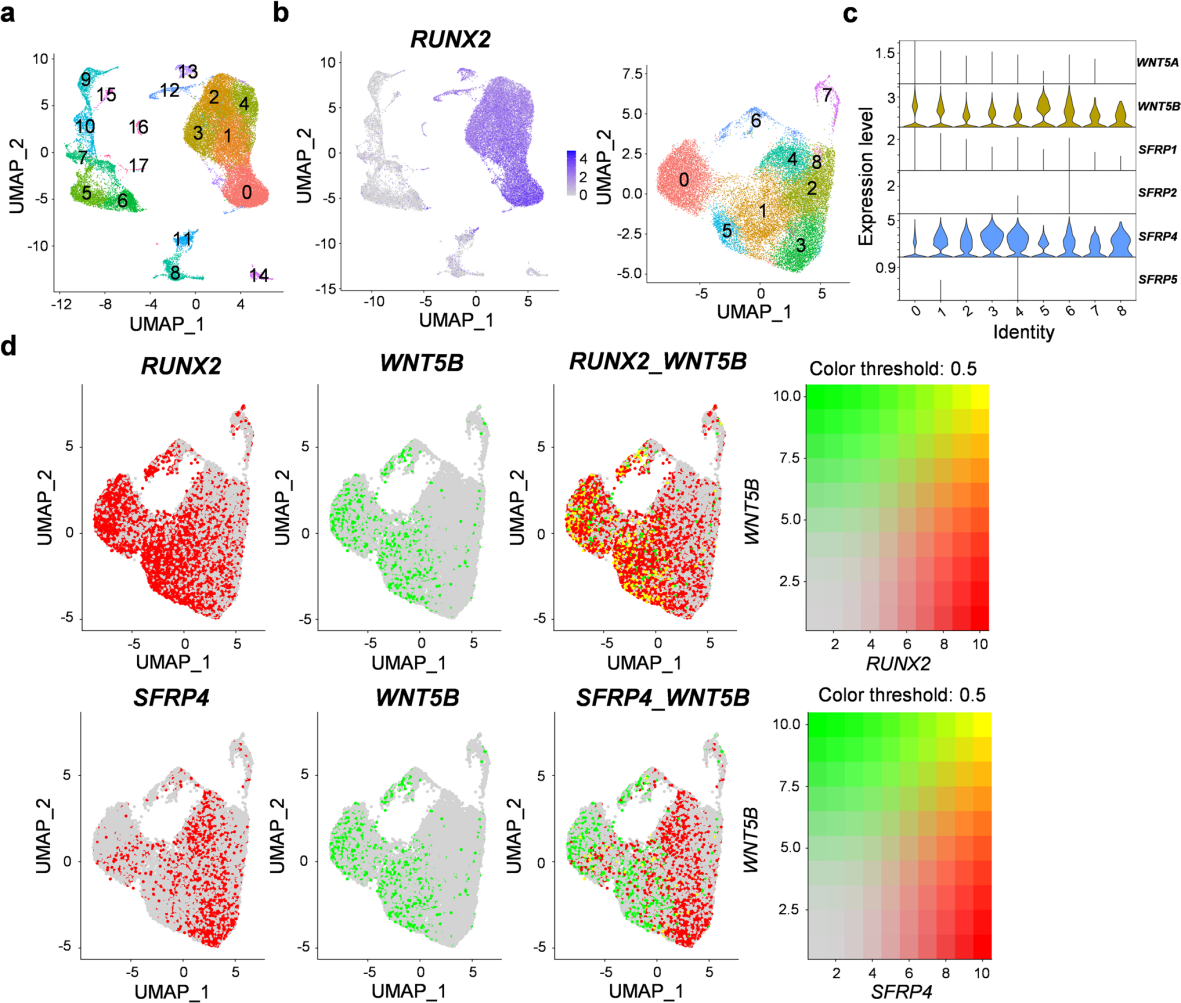
GCTB expresses *SFRP4* and *WNT5B.* **a** UMAP plot of reanalyzed GCTB scRNA-seq data integrating four species from GSE149211. Eighteen subpopulations are identified based on gene expression profiles. **b** Left: *RUNX2* expression in UMAP plot shown in (**a**). Right: *RUNX2* expressing cells were extracted from the left panel, and nine clusters were newly detected as *RUNX2* expressing subpopulations. **c** Expression of osteoblastogenesis inhibitory factors, which potentially inhibit Wnt/β-catenin signaling, based on GCTB scRNA-seq data. **d** Expression of *RUNX2* and *WNT5B* (upper) or *SFRP4* and *WNT5B* (lower) visualized in *RUNX2*-expressing subpopulations

To comprehensively assess expression of potential inhibitory factors secreted by GCTB cells, we reanalyzed scRNA-seq data deposited in publicly available databases (Fig. 2a). Given that GCTB cells express *RUNX2* (Fig. 1b), we extracted *RUNX2-*positive clusters (Fig. 2b) and monitored expression of factors known to inhibit Wnt/β-catenin signaling [16–18], which is essential for osteoblast differentiation, in those cell populations. We found that *RUNX2*-expressing cells expressed *SFRP4* and *WNT5B* (Figs. 2c and Supplemetary Fig. 2). To determine whether the same cells co-expressed both *SFRP4* and *WNT5B*, we visualized both genes using FeaturePlot and assessed potential overlap (Fig. 2d). In the analysis shown in Fig. 2d, each dot represents a cell, and yellow dots indicate cells highly expressing both *SFRP4* and *WNT5B*. Using that approach, we identified cells co-expressing both *RUNX2* and *WNT5B,* but observed few cells expressing both *SFRP4* and *WNT5B* (Fig. 2d). We then reanalyzed previously reported scRNA-seq data for osteoblast-lineage cells (Supplementary Fig. 3a) and found that during normal osteoblast differentiation, both *SFRP4* and *WNT5B* tended to be mainly expressed at late differentiation stages (Supplementary Fig. 3b) albeit in distinct cell populations (Supplementary Fig. 3c).

**Fig. 3:**
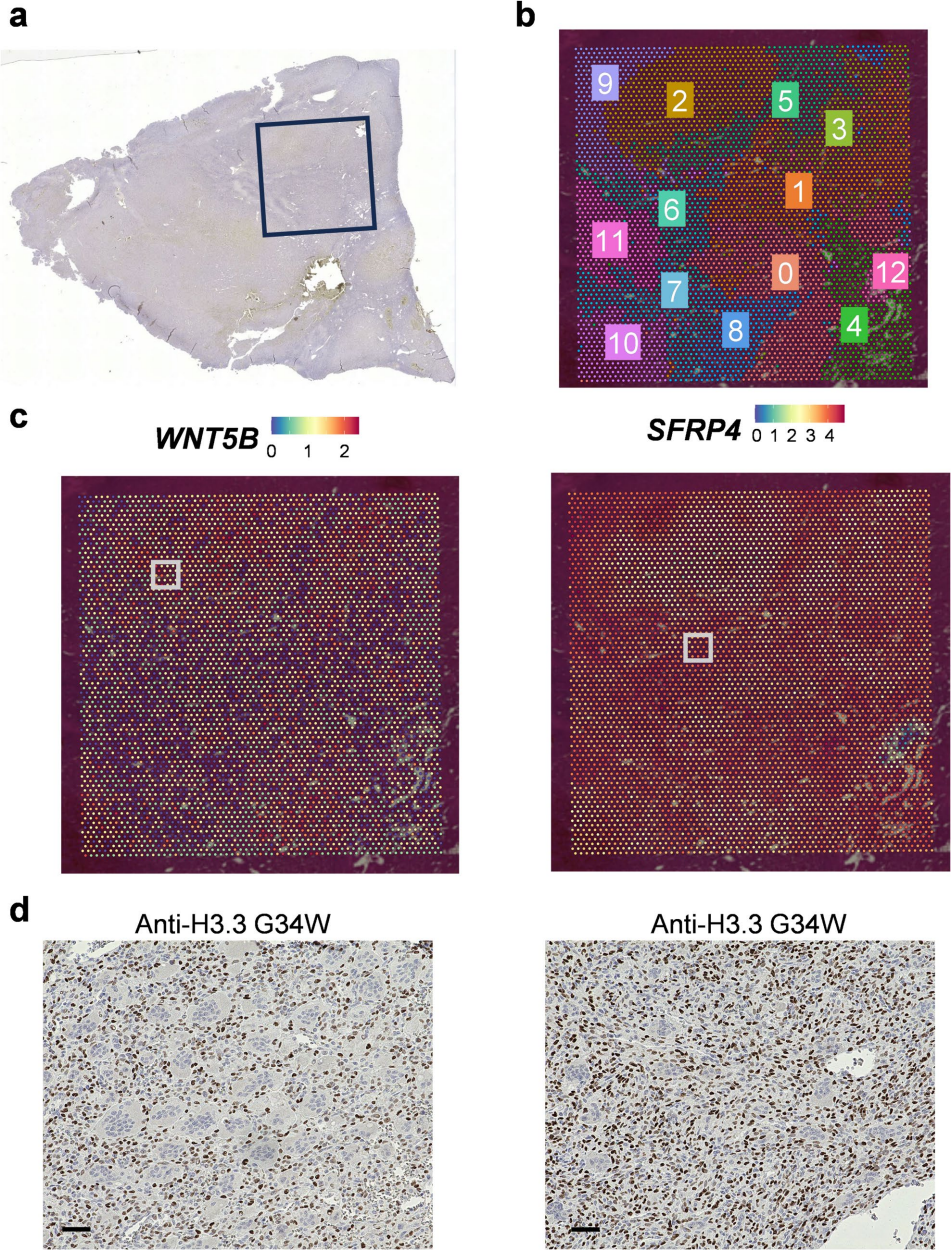
Spatial transcriptome analysis (Visium) of a GCTB section. **a** A paraffin section was prepared from surgically dissected GCTB and stained with hematoxylin. A serial section was used for spatial transcriptome analysis by Visium. The square indicates the area analyzed by Visium. **b** Thirteen clusters were identified by Visium based on gene expression. **c** and **d** Shown is the expression of *WNT5B* or *SFRP4*, both of which are Wnt/β-catenin signaling inhibitors, in Visium (**c**). Gray squares in (**c**) indicate regions of interest, which are shown below at higher magnification (**d**). The sections, separated from the same paraffin block used for Visium analysis, were stained with rabbit anti-H3.3 G34W followed by HRP-conjugated goat anti-rabbit Ig’ antibodies. Sections were then treated with DAB. Nuclei were stained with hematoxylin, and observed under a microscope. **d** Bar = 50 µm

We next examined *SFRP4* and *WNT5B* expression patterns using Visium. To do so, we prepared sections from paraffin blocks of surgical GCTB specimens excised at our hospital and performed spatial transcriptomics analysis on regions indicated by squares in Fig. 3a. Analysis of transcript profiles grouped the tissue into 13 clusters (Fig. 3b). Immunohistochemical staining of sections from the same paraffin block using an antibody against H3.3 G34W—a GCTB marker—revealed diffuse distribution of GCTB stromal cells within areas analyzed (Supplementary Fig. 4). Visium analysis revealed distinct *SFRP4* and *WNT5B* expression patterns in the section (Fig. 3c). We then performed immunohistochemical staining of separate sections from the same paraffin block used for Visium analysis using an anti-H3.3 G34W antibody (Fig. 3d). Taken together, the immunohistochemical staining and Visium SpatialFeaturePlot analyses indicated that regions with high *WNT5B* expression tended to be enriched with multinucleated giant cells, as indicated by *ACP5*, while *SFRP4* was expressed in areas rich in monocyte-like mononuclear cells, as indicated by *CD14* (Fig. 3c, d, and Supplementary Fig. 5a). The heatmap analysis demonstrated that *WNT5B* tended to be highly expressed in clusters exhibiting high expression of *ACP5*, whereas *SFRP4* tended to exhibit higher expression levels in clusters with high *CD14* expression (Supplementary Fig. 5b). Reanalyzing GCTB scRNA-seq data suggested that clusters expressing *ACP5* or *CD14* differed from clusters expressing *WNT5B* or *SFRP4* (Supplementay Fig. 6).

### WNT5B is highly expressed in GCTB1s

**Fig. 4:**
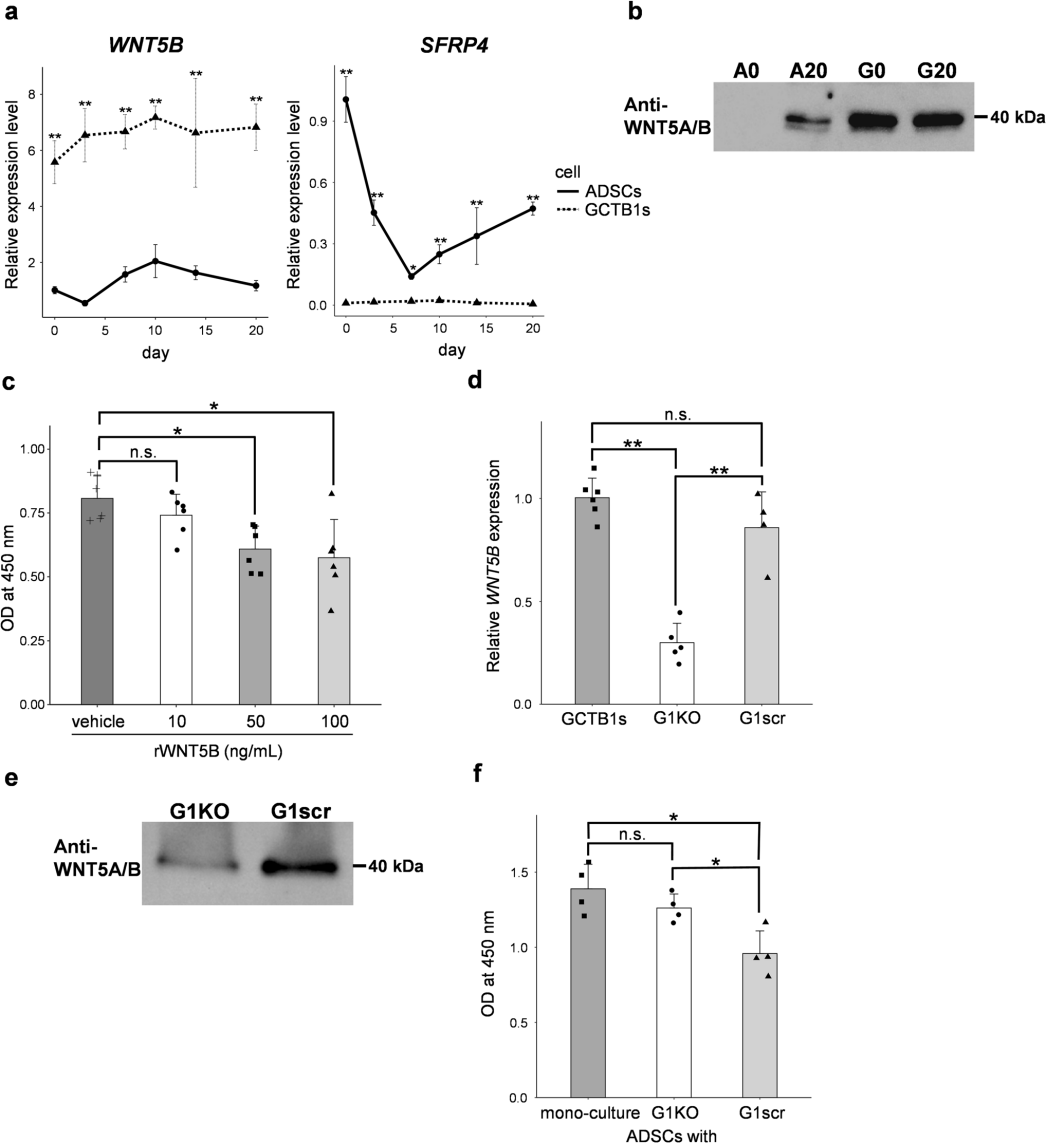
*WNT5B* knockout in GCTB1s rescues ADSC mineralization in a co-culture system. **a** ADSCs and GCTB1s were cultured separately in osteogenic induction medium and *WNT5B* or *SFRP4* expression in both was analyzed by qPCR on days 3, 7, 10, 14, or 20 of osteogenic induction. Data represents mean *WNT5B* or *SFRP4* expression relative to *β-actin* (*ACTB*) ± SD (n = 6, Mann–Whitney *U* test: **p < 0.01, *p < 0.05). **b** ADSCs and GCTB1s were cultured separately in osteogenic induction medium, and supernatants of each culture were collected on days 0 or 20 (A0, ADSC supernatant on day 0; A20, ADSC supernatant on day 20; G0, GCTB1 supernatant on day 0; G20, GCTB1 supernatant on day 20). Expression of WNT5A/B protein was then analyzed by Western blot. **c** ADSCs were cultured in osteogenic induction medium with or without various concentrations of recombinant WNT5B (rWNT5B) protein for 21 days. All samples were then stained with Alizarin Red S, which was extracted in 5% formic acid and assessed for absorbance at 450 nm to quantify mineralization. Data represents mean absorbance ± SD (each; n = 6, Welch’s test: *p < 0.05). **d** *WNT5B* knockout GCTB1s (G1KO) and control GCTB1s (GCTB1 scramble, G1scr) were established, and *WNT5B* expression in both was analyzed by qPCR. Data represents mean *WNT5B* expression relative to *β-actin* (*ACTB*) ± SD (GCTB1s, *n* = 6; G1KO, *n* = 5; G1scr, *n* = 4. Welch’s test: **p < 0.01). **e** G1KO or G1scr were cultured separately for 24 h in DMEM without FBS. Supernatants were then collected, and expression of WNT5A/B protein was analyzed by Western blot. **f** ADSCs were co-cultured with G1KO or G1scr in osteogenic induction medium in a horizontal co-culture system for 18 days. ADSC mono-culture with osteogenic induction medium served as a control culture. All samples were then stained with Alizarin Red S, which was extracted in 5% formic acid and assessed for absorbance at 450 nm to quantify mineralization. Data represents mean absorbance ± SD (each; n = 4, Welch’s test: *p < 0.05)

Next, to determine the expression of *WNT5B* and *SFRP4* in GCTB1s and ADSCs during osteoblastic differentiation, we cultured GCTB1s and ADSCs in osteogenic induction medium and analyzed *WNT5B* and *SFRP4* expression over a 20-day period by qPCR (Fig. 4a). *WNT5B* expression was significantly higher in GCTB1s than in ADSCs through the entire culture period, while in GCTB1s *SFRP4* expression was significantly lower than in ADSCs (Fig. 4a). We then cultured GCTB1s and ADSCs separately in osteogenic induction medium and performed Western blot analysis on culture supernatants collected at 0 or 20 days after initiation of differentiation (Fig. 4b). WNT5A/B protein levels in supernatants from GCTB1s at 0 and 20 days after the initiation of osteoblastic differentiation induction were higher than those in supernatants from ADSCs at 20 days after differentiation induction (Fig. 4b). *WNT5A* expression was comparable or slightly higher in ADSCs than in GCTB1s (Supplementary Fig. 7). Therefore, we interpret the band detected by the anti-WNT5A/B antibody in Western blot analysis of GCTB1s culture supernatants to be primarily WNT5B. Using the same paraffin blocks of tumor tissue used for Visium analysis, we observed diffuse staining for WNT5A/B (Supplementary Fig. 8a, b).

WNT5B has been reported to inhibit osteoblast differentiation [31]. In addition, we found that osteoblastic differentiation of ADSCs was significantly inhibited by adding recombinant WNT5B protein to the ADSC culture (Fig. 4c). To determine whether GCTB1s inhibit osteoblastic differentiation of ADSCs by secreting WNT5B, we generated *WNT5B* knockout GCTB1s (G1KO) using the CRISPR/Cas9 system. GCTB1s transfected with a scramble vector (G1scr) served as controls. G1scr exhibited *WNT5B* expression comparable to that of parental GCTB1s, while G1KO showed significantly lower *WNT5B* transcript levels than controls (Fig. 4d). Western blot analysis also showed decreased WNT5B protein levels in culture supernatants from G1KO relative to G1scr (Fig. 4e).

We then performed horizontal co-culture of ADSCs with either G1KO or G1scr. In ADSCs/G1scr co-cultures, ADSCs mineralization was significantly inhibited compared to ADSCs in mono-culture, while in contrast, ADSCs in horizontal co-culture with G1KO exhibited mineralization comparable to ADSCs in mono-culture and significantly higher than mineralization seen in ADSCs in horizontal co-culture with G1scr (Fig. 4f). These results suggest that WNT5B secreted by GCTB1s inhibits ADSC mineralization.

### Culture supernatants from ADSCs at a late stage of osteoblastic differentiation induce mineralization in GCTB1s

**Fig. 5:**
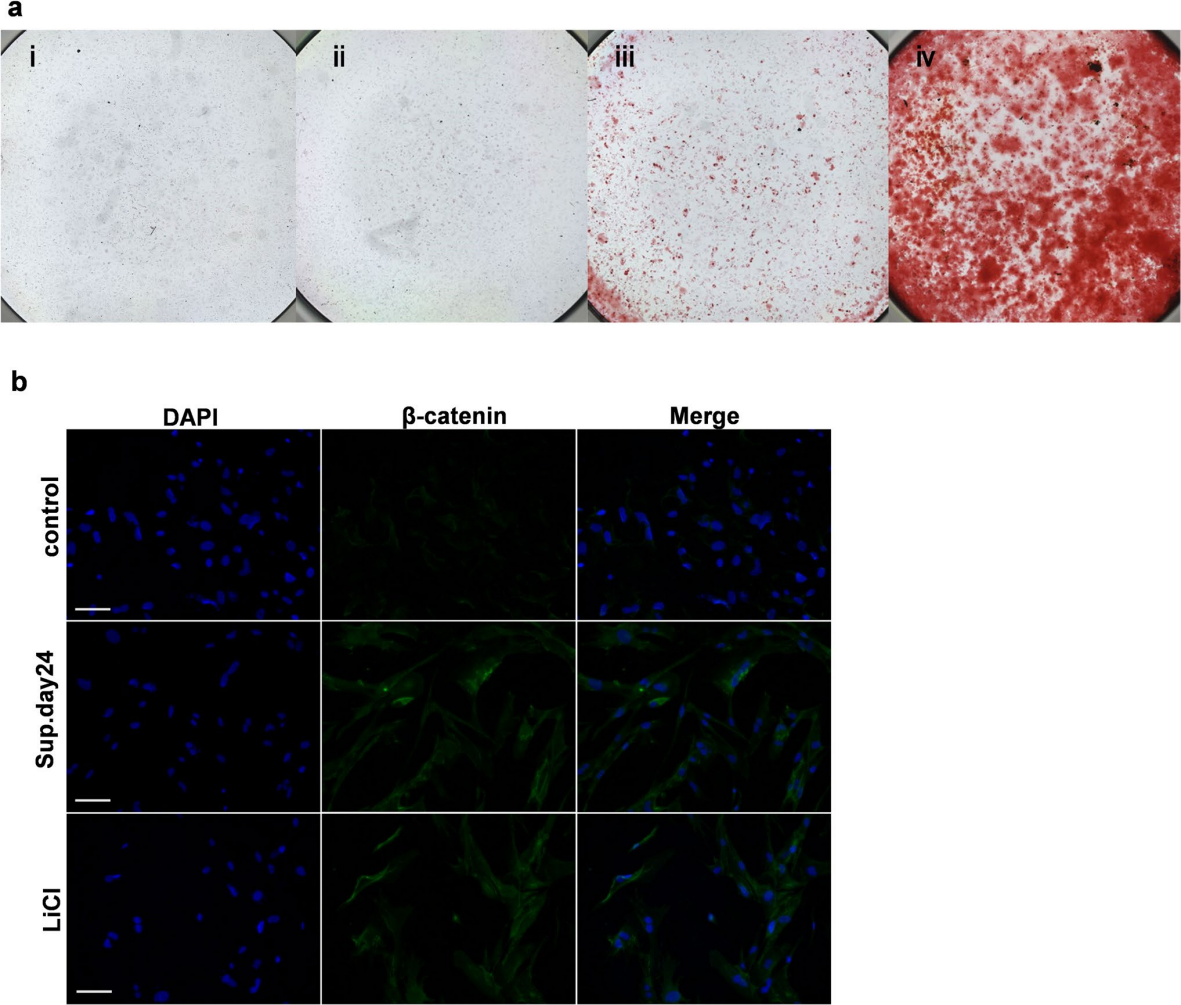
ADSC supernatants induce GCTB1 mineralization. **a** ADSCs were cultured in osteogenic induction medium for 7, 21, or 24 days, and then culture supernatants were collected and applied to cultures of GCTB1s. After 24 h, the medium was changed to osteogenic induction medium every 3 days for a total of 8 days. Alizarin Red S staining was then performed at day 8, and cells were observed under a microscope. ii, 7-day supernatants; iii, 21-day supernatants; and iv, 24-day supernatants. i, control GCTB1s cultured in osteogenic induction medium only. **b** GCTB1s were cultured in osteogenic induction medium (control) or either supernatant of ADSCs cultured 24 days in osteogenic induction medium (Sup.day24) or 50 mM lithium chloride (LiCl) on chamber slides. Five minutes after Sup.day24 or LiCl addition, cells were fixed with 4% PFA, stained with rabbit anti-non-phospho (active) β-catenin antibody followed by Alexa488-conjugated anti-goat Ig’ antibody, and observed under a fluorescence microscope. DAPI served as a nuclear marker. Bar = 100 µm

To analyze the effects of supernatants from differentiated osteoblasts on GCTB cells, we collected culture supernatants from both early and late stages of ADSC osteoblastic differentiation, namely, on days 7, 21, and 24. After centrifuging supernatants to remove dead cells, we used them to culture GCTB1s for 24 h and then replaced that medium with standard osteogenic induction medium, while control GCTB1s were cultured in standard osteogenic induction medium without supernatants. Eight days later, to monitor mineralization, we then performed Alizarin Red S staining of all samples. We observed no mineralization in either control GCTB1s or GCTB1s treated with culture supernatants from early stages. However, we did observe mineralization in GCTB1s treated with supernatants collected from ADSCs on culture day 21 and day 24 post-differentiation (Fig. 5a).

Next, we performed qPCR of the GCTB1s described above for *CTNNB1*, which encodes β-catenin, 6 h after adding the supernatants described above (Supplementary Fig. 9). In GCTB1s cultured with supernatants from days 21 or 24 ADSCs, *CTNNB1* transcript levels were significantly higher than levels seen in GCTB1s cultured in either standard osteogenic induction medium (controls) or supernatants collected from ADSCs at day 7 of differentiation (Supplementary Fig. 9). When we performed immunofluorescence staining for active β-catenin 5 min after adding supernatant or control (Fig. 5b), GCTB1s treated with medium from ADSCs at day 24 of differentiation showed more intense staining than control (Fig. 5b). In addition, as a positive control representing Wnt activation, we cultured GCTB1s with osteogenic induction medium containing 50 mM LiCl, which inhibits GSK3β and activates β-catenin. Those cells also showed more intense active β-catenin staining than control (Fig. 5b).

## Discussion

GCTB tumors contain giant cells, namely, osteoclasts that promote bone resorption and destruction [8, 32]. GCTB is a tumor of stromal cells surrounding osteoclasts; thus, its characteristic giant cells are osteoclasts induced by the tumor [3, 33]. In normal bone metabolism, coupling of osteoclast-mediated bone resorption reciprocally activates osteoblast-mediated bone formation, and thus increased bone resorption leads to increased bone formation [13]. However, in GCTB, radiolucent areas are often visible on X-rays and CT scans, and activation of bone formation is not frequently seen [34]. Some attribute this outcome to the fact that bone resorption activity in GCTB is extremely high, preventing detection of significant levels of subsequent bone formation and resulting in the radiolucent areas seen in X-rays and CT [8]. Our findings suggest a different interpretation, namely, that GCTB cells can inhibit bone formation via WNT5B secretion.

Adult T-cell leukemia (ATL) is characterized by pathologically active bone resorption [35]. ATL cells infiltrating the bone marrow induce osteoclast differentiation and bone resorption, sometimes leading to fatal hypercalcemia [36]. We previously reported that ATL tumor cells express RANKL, a cytokine essential for osteoclast differentiation [37]. It has also been reported that GCTB cells express RANKL [8, 23].

Paget's disease of the bone is also a well-known disorder characterized by pathologically increased bone resorption [38]. In that case, increased bone resorption is accompanied by increased bone formation, leading to abnormally high bone metabolism and resulting in bone deformities and reduced bone strength [39]. By contrast, in multiple myeloma, bone resorption is reportedly increased, while bone formation is suppressed [40] due to secretion by multiple myeloma cells of DKK1 or activin A, both of which are inhibitors of bone formation [41, 42].

Here, we demonstrated that GCTB cells inhibit mineralization by expressing WNT5B. *WNT5B* is one of 19 Wnt family genes in humans and encodes a factor that inhibits osteoblast activity [31, 43]. Unfortunately, we could not assess whether WNT5B expression by GCTB cells impacted tumor growth, although we attempted to do so by establishing a patient-derived xenograft (PDX) model by transplanting GCTB patient-derived cells (PDCs) into the peritoneal cavity and bone marrow of immunodeficient mice*.* We were unable to establish this model and note that others report similar difficulties in establishing GCTB PDX models [44]. We also show here that GCTB cells express *RUNX2*, a transcription factor essential for osteoblast differentiation, and thus, GCTB is a tumor of mesenchymal stem cell lineage. This finding suggests that GCTB cells may inhibit the activity of competing cells by suppressing the differentiation of surrounding osteoblasts via paracrine WNT5B (Supplementary Fig. 10). Indeed, we demonstrated that soluble factors secreted by mature osteoblasts promote the mineralization of GCTB cells (Fig. 5a), suggesting that GCTB may remain undifferentiated by suppressing the differentiation of surrounding osteoblasts (Supplementary Fig. 10). Interestingly, we found that *WNT5B* expression in GCTB cells significantly increased at 6 h after the addition of culture supernatant from ADSCs at the late stage of osteoblastic differentiation induction (Supplementary Fig. 11). This suggests that GCTB cells may reactively upregulate *WNT5B* expression to suppress differentiation induction signals. On the other hand, GCTB cells with *WNT5B* knockdown also failed to induce mineralization when cultured in osteogenic induction medium (Supplementary Fig. 12). This suggests that GCTB cells may express other osteoblast differentiation inhibitory factors besides WNT5B. Indeed, we found that GCTB cells express *DKK3*, another osteoblast differentiation inhibitory factor [45], at significantly higher levels than ADSCs (Supplementary Fig. 13a). In GCTB scRNA-seq data, similar to *WNT5B*, *DKK3* expression was observed in the *RUNX2*-rich cluster (Supplementary Fig. 13b). These results suggest that GCTB cells maintain their undifferentiated state by expressing several osteoblast differentiation inhibitory factors.

We showed that *WNT5B* tends to be highly expressed in the preosteoblast cluster, while *SFRP4* is primarily expressed in the mature osteoblast cluster (Supplementary Fig. 3b, c). In addition, we showed that GCTB cells highly express *WNT5B*, whereas *SFRP4* expression is low (Fig. 4a). The absence of *SFRP4* expression in GCTB cells aligns with the previous report [8]. Our Visium analysis of GCTB revealed expression of both *WNT5B* and *SFRP4* in the tumor tissue (Fig. 3c). Considering these findings collectively, we consider that GCTB cells may originate from *WNT5B*-positive cells within the osteoblast differentiation lineage that have become tumorigenic (Supplementary Fig. 10). Furthermore, the *SFRP4*-positive cells identified by GCTB's Visium analysis are considered to be normal osteoblast-lineage cells expressing *SFRP4* within the tumor tissue. Although the distinct roles of *WNT5B* and *SFRP4* remain unclear, their expression patterns change during normal osteoblast differentiation, suggesting they likely function to suppress differentiation at different stages.

WNT5B is most closely related to WNT5A, and both proteins are known to activate noncanonical Wnt signaling [46]. Relevant to Wnt5a, it is reported that Wnt5a secreted from osteoblasts activates osteoclast formation via Ror2, a receptor expressed on osteoclasts [47]. Wnt5a-deficient mice exhibit skeletal abnormalities such as malformations of the body axis and limbs [48]. Both Wnt5a and Wnt5b were reportedly accelerated osteoclastogenesis [47, 49], whereas Wnt5a promoted osteoblast differentiation while Wnt5b may inhibit it [31, 50]. We show here that GCTB expresses WNT5B but not WNT5A, and WNT5B may function in GCTB tumor growth.

## Supplementary Information

Below is the link to the electronic supplementary material.

Supplementary file1 (PDF 1462 KB)

Supplementary file2 (PDF 251 KB)

## Data Availability

All data generated or analyzed during this study are included in this published article (and its Supplementary Information files). The Visium data are available from the Gene Expression Omnibus (GEO) repository with the accession code GSE311123.
