# Supplementary material for: Giant cell tumor of bone inhibits osteoblastogenesis via WNT5B
Source: J Bone Miner Metab. 2026 Mar 23;44(4):455–68. doi: 10.1007/s00774-026-01713-3 (PMC13469380; doi:10.1007/s00774-026-01713-3)

Supplementary Fig. 1

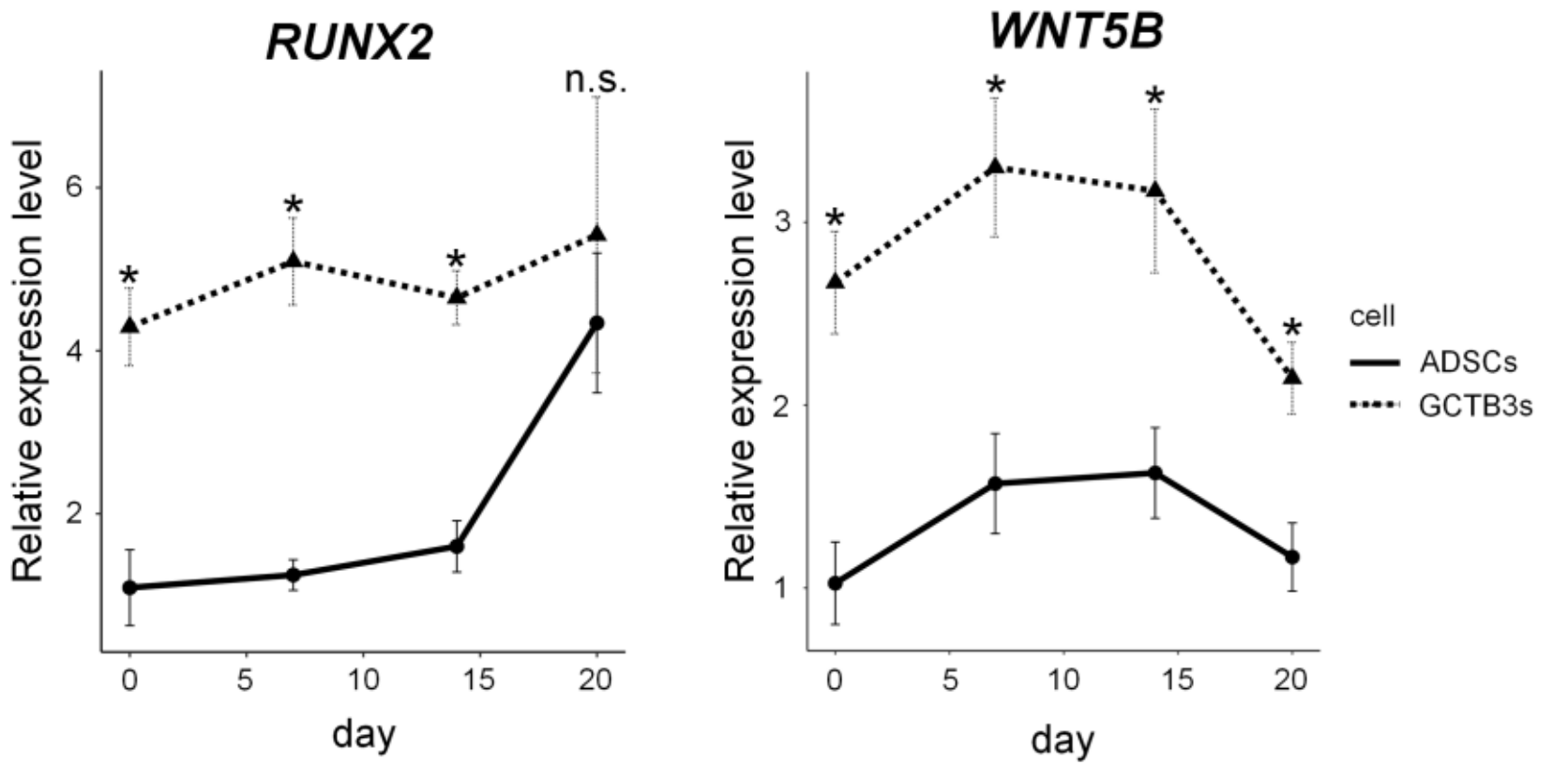

Supplementary Fig. 2

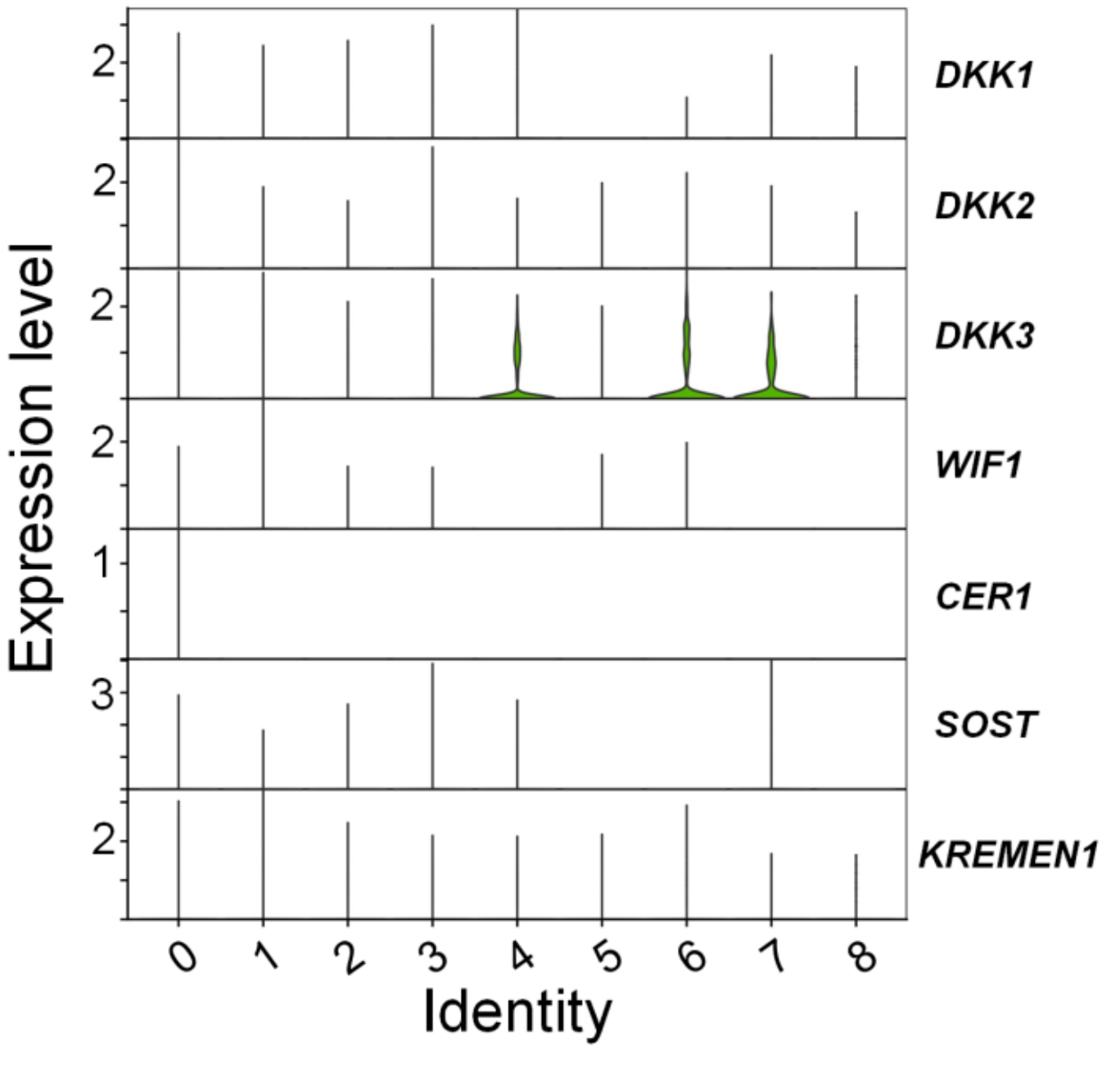

Supplementary Fig. 3

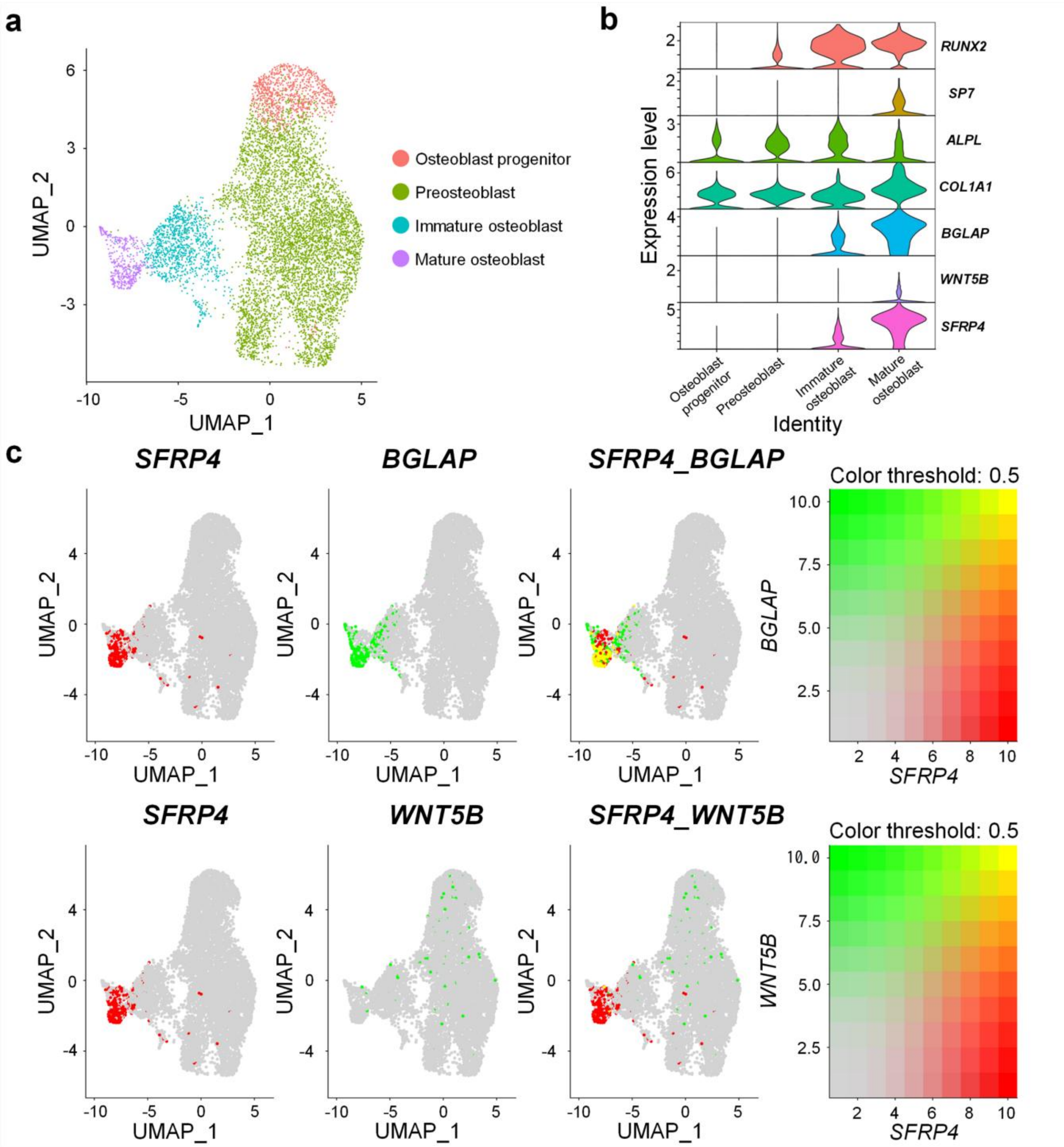

Supplementary Fig. 4

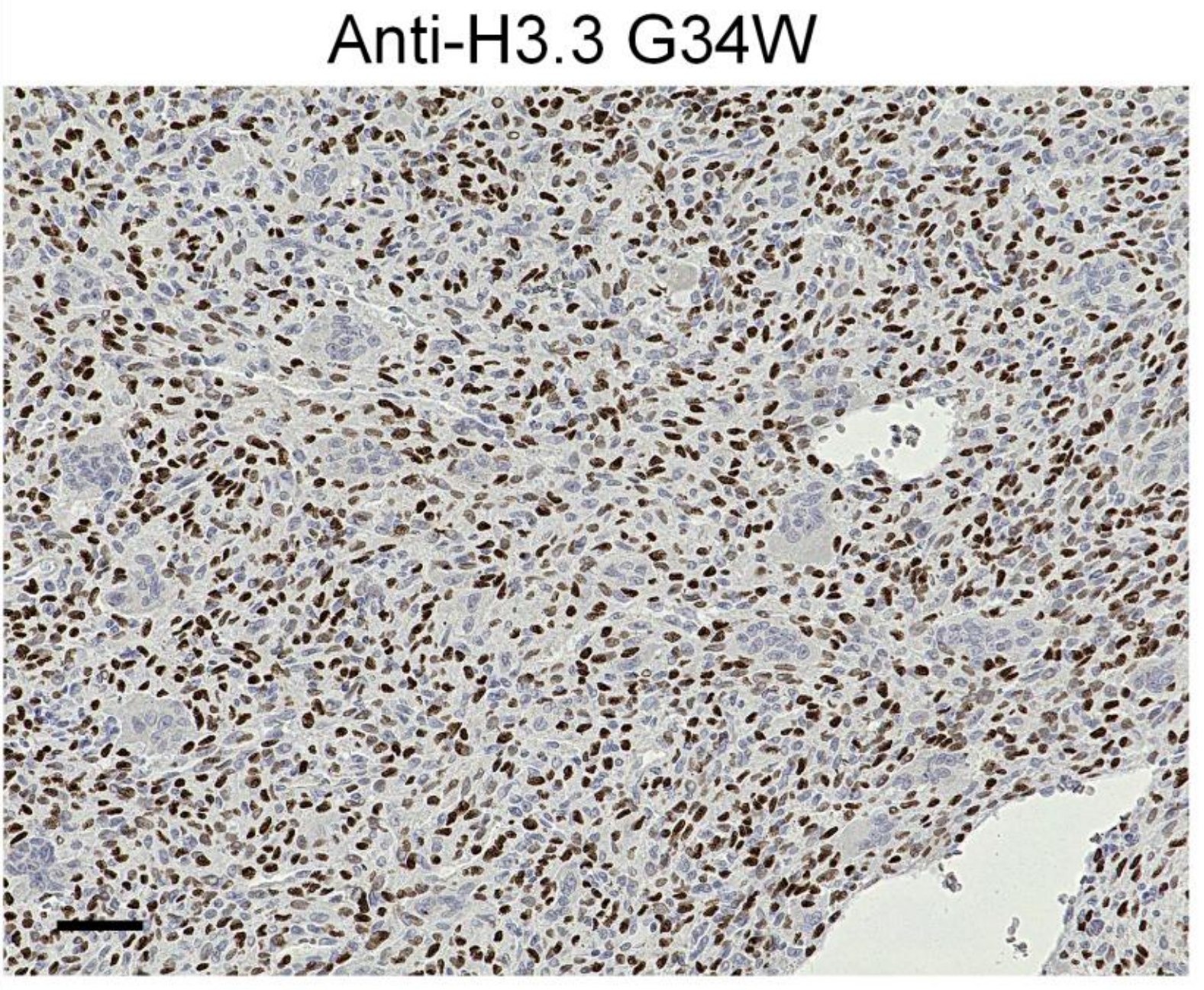

Supplementary Fig. 5

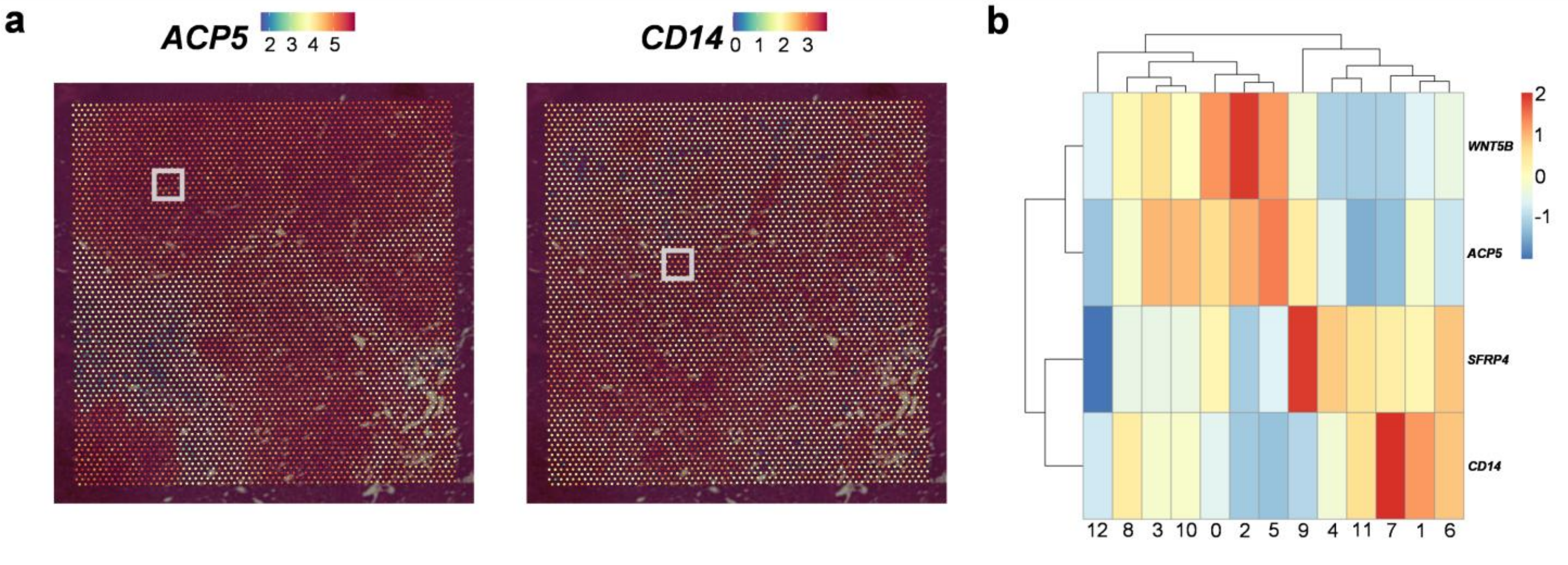

Supplementary Fig. 6

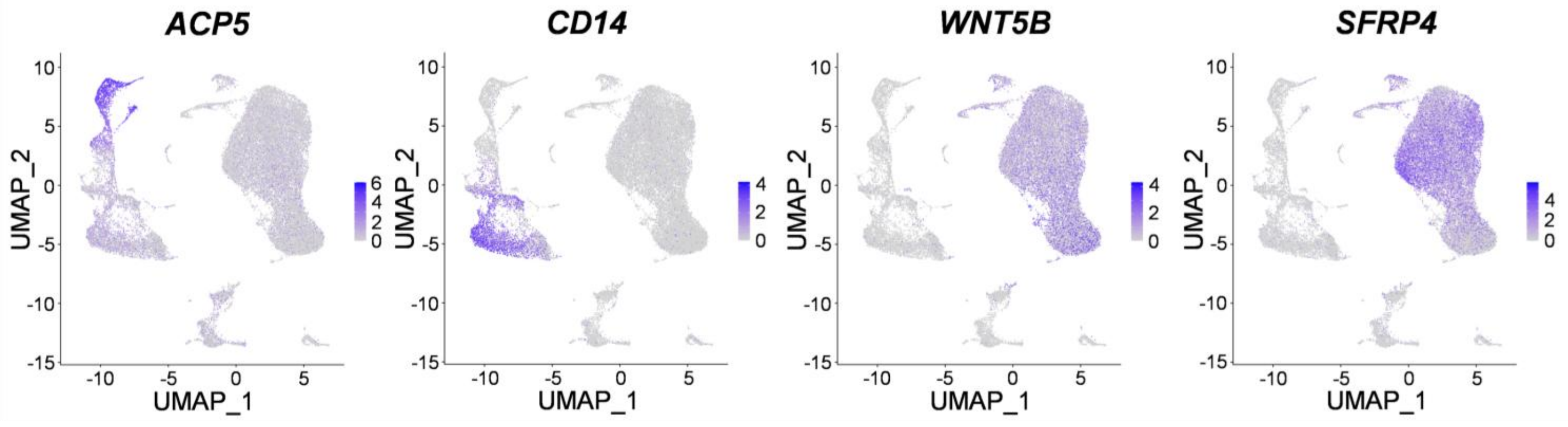

Supplementary Fig. 7

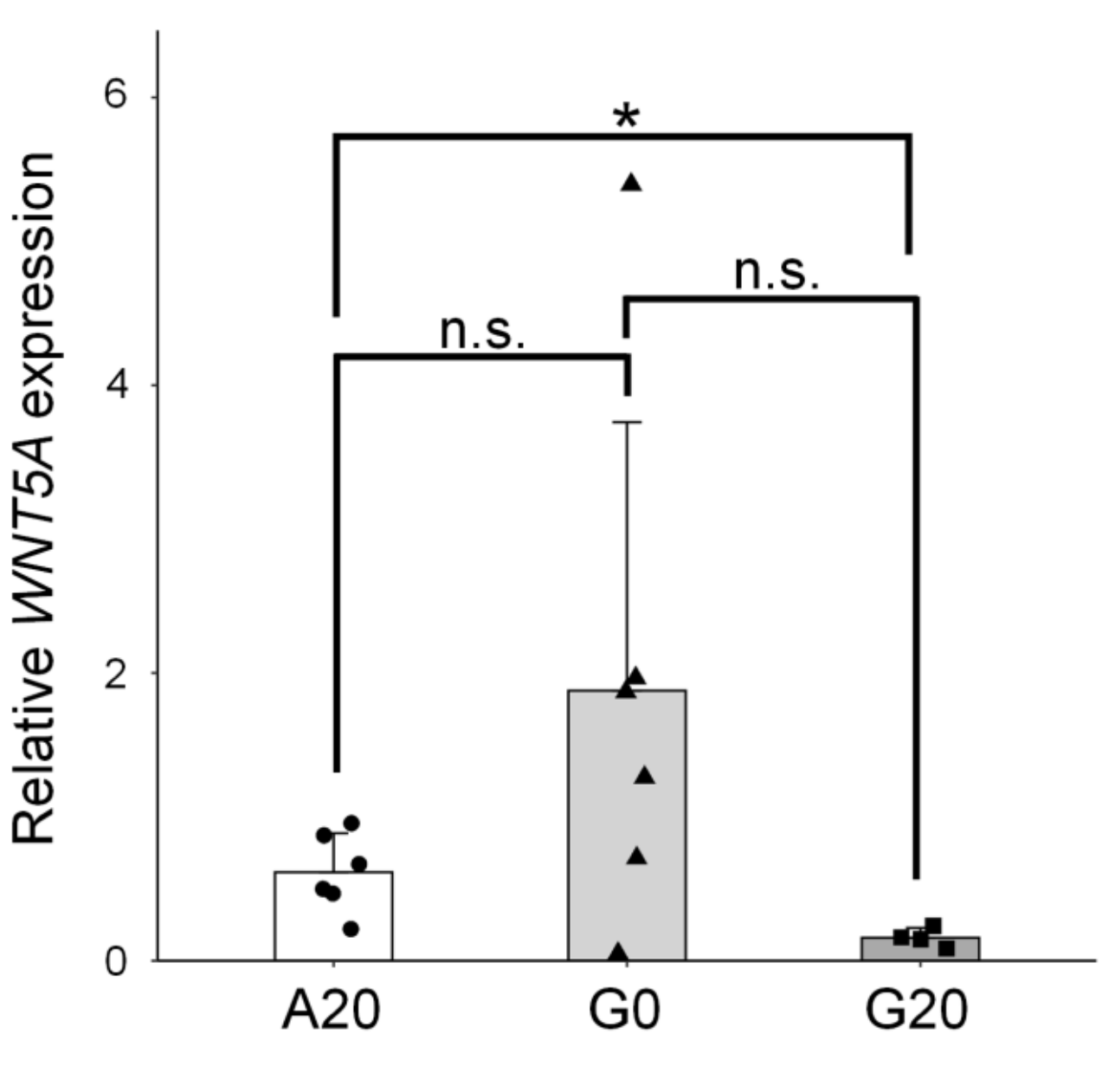

Supplementary Fig. 8

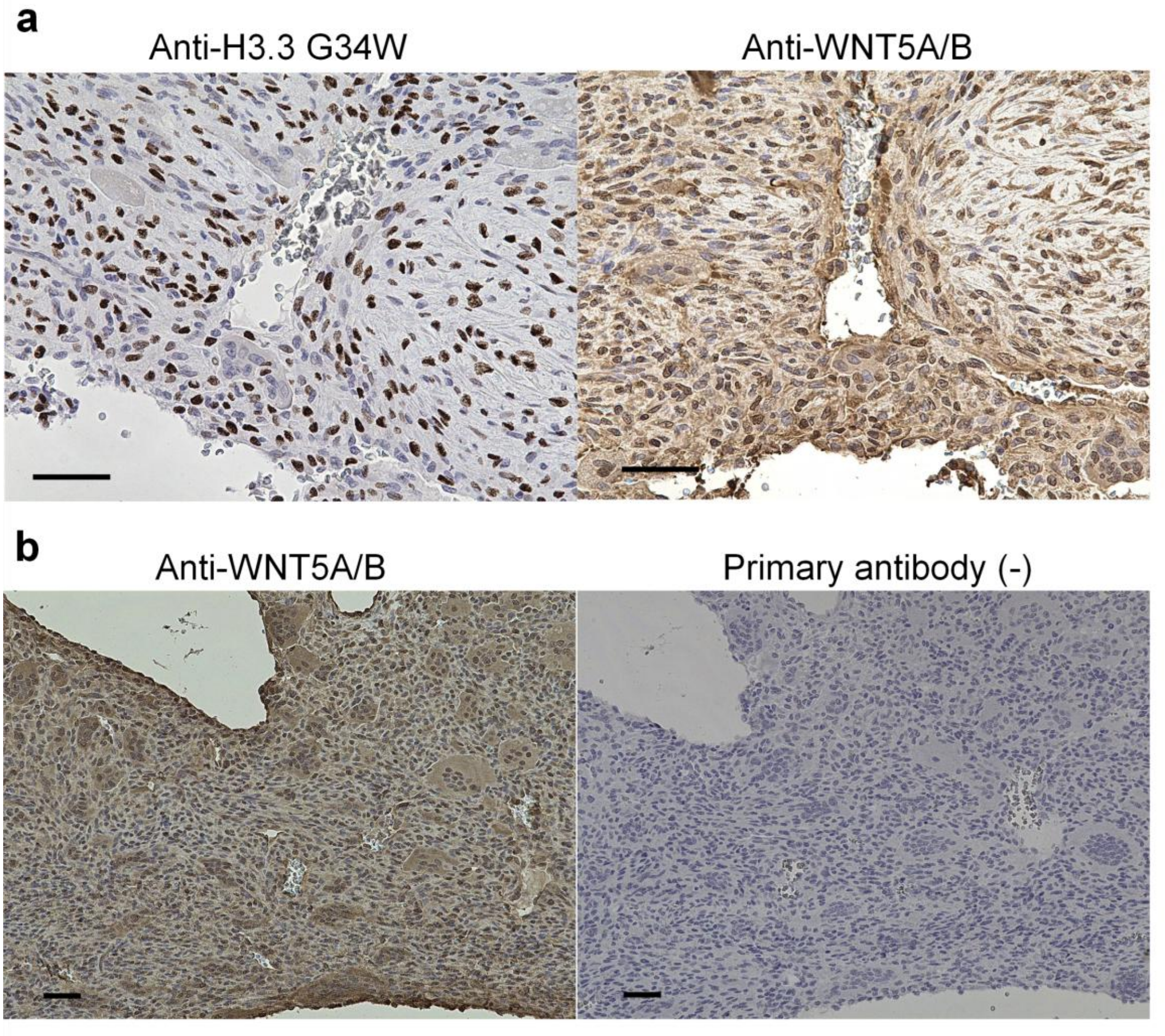

Supplementary Fig. 9

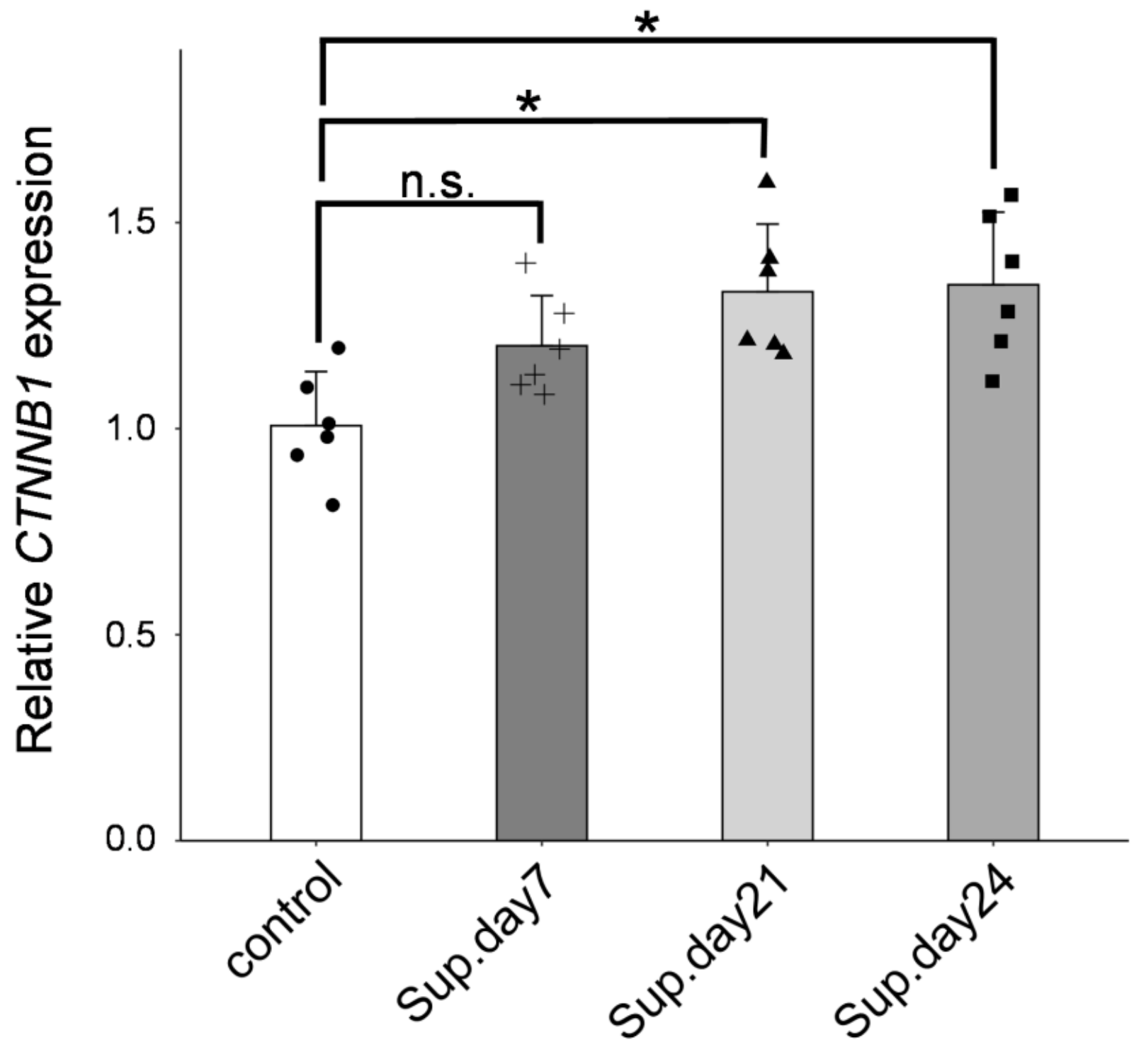

Supplementary Fig. 10

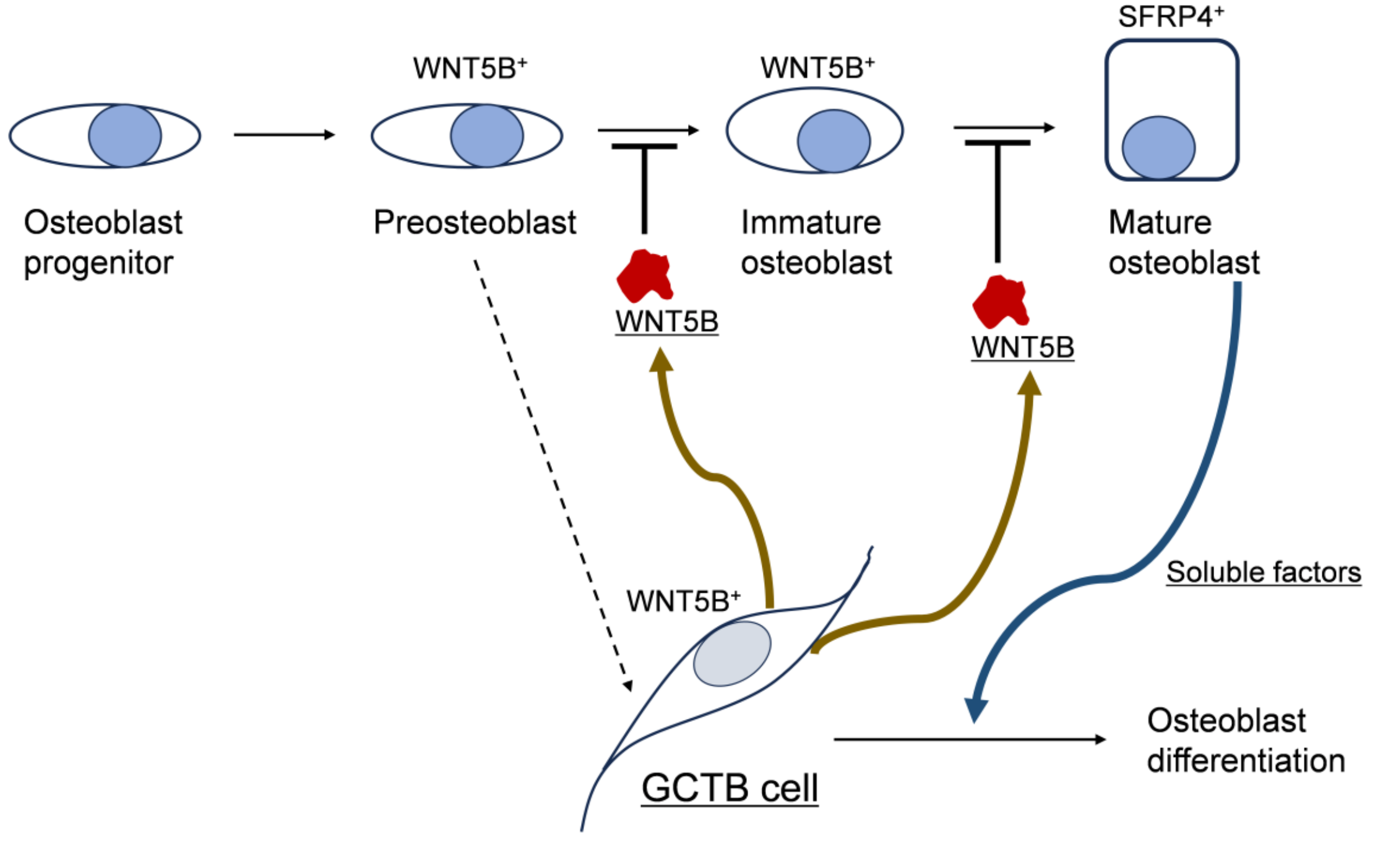

Supplementary Fig. 11

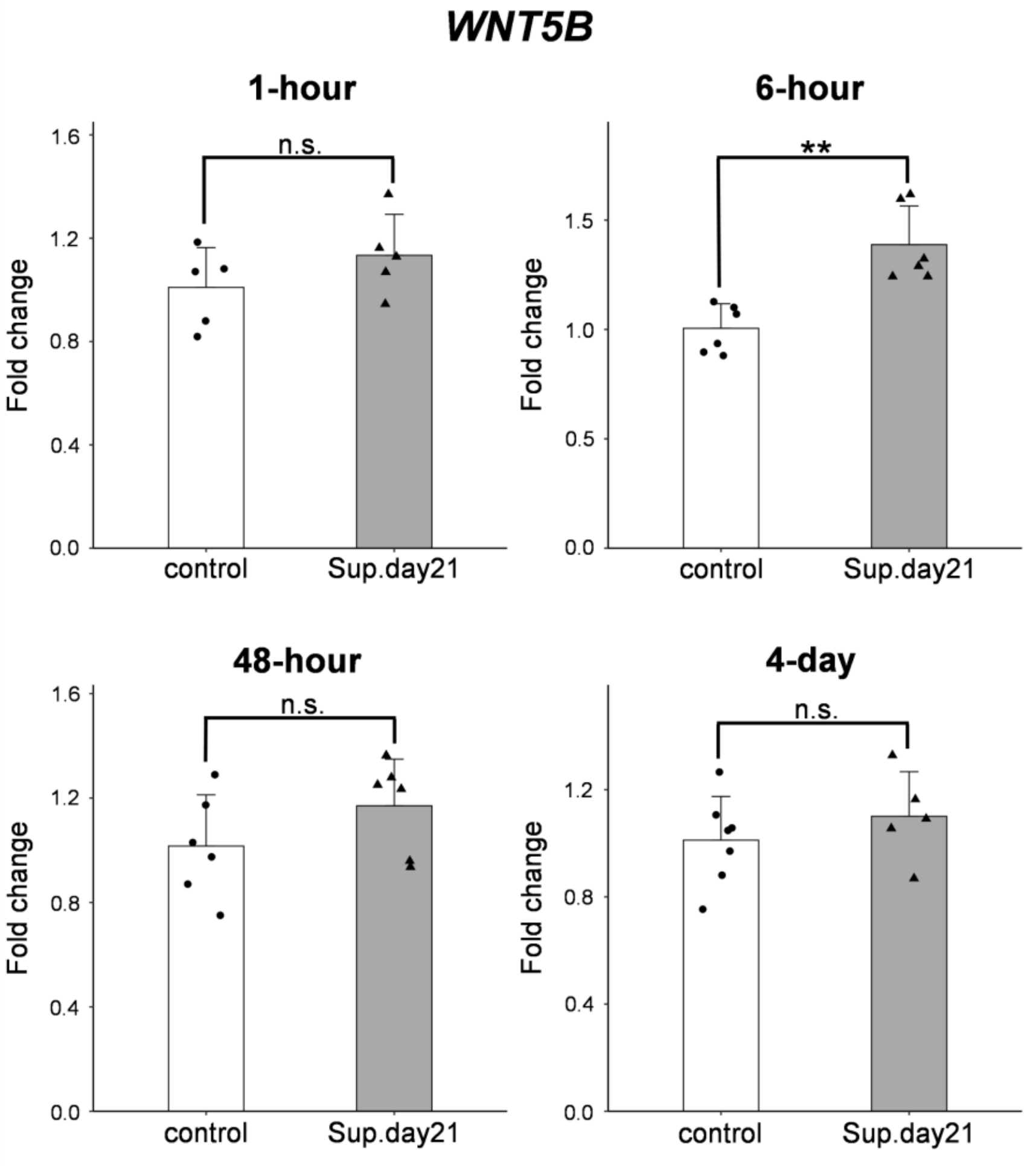

Supplementary Fig. 12

G1KO

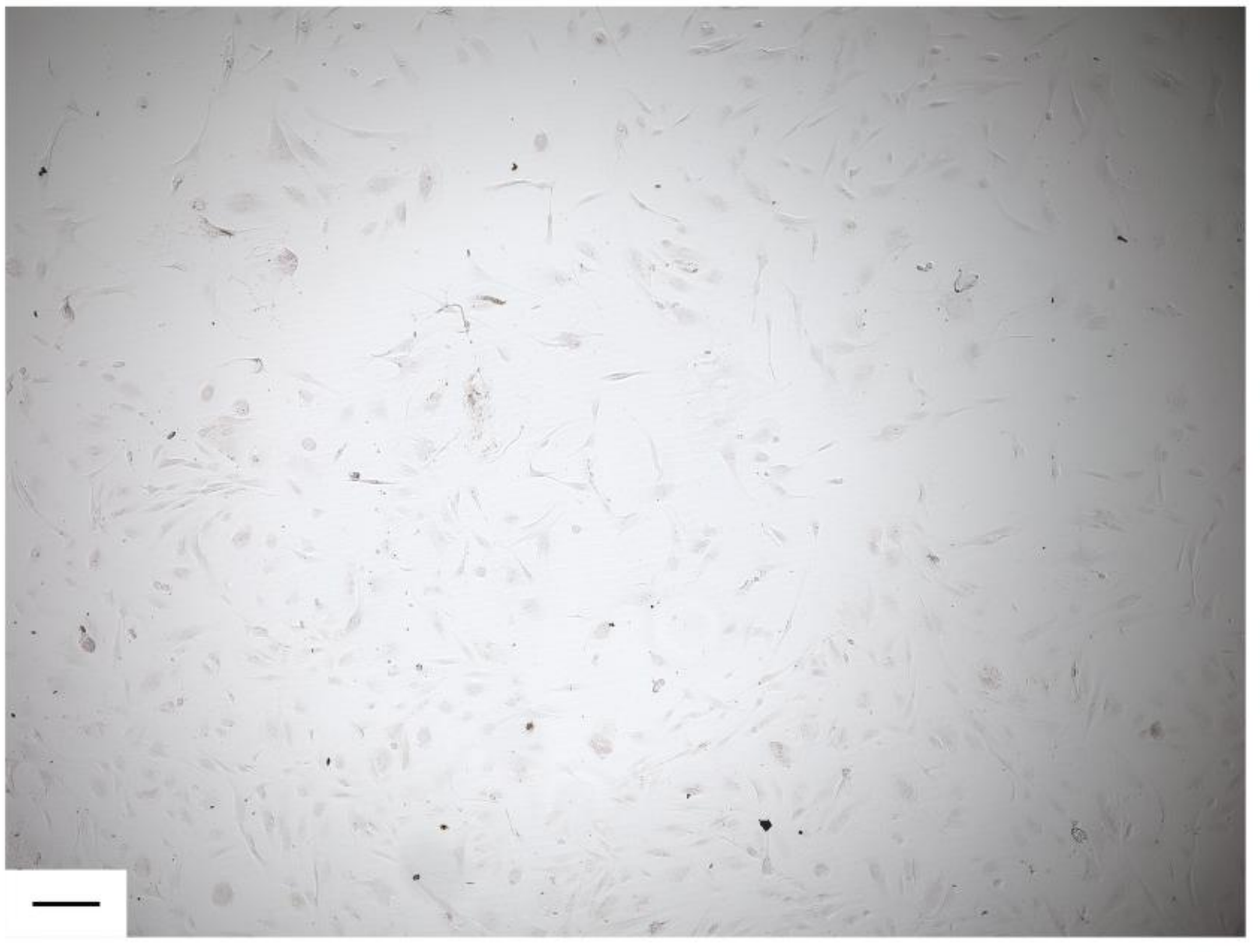

Supplementary Fig. 13

a

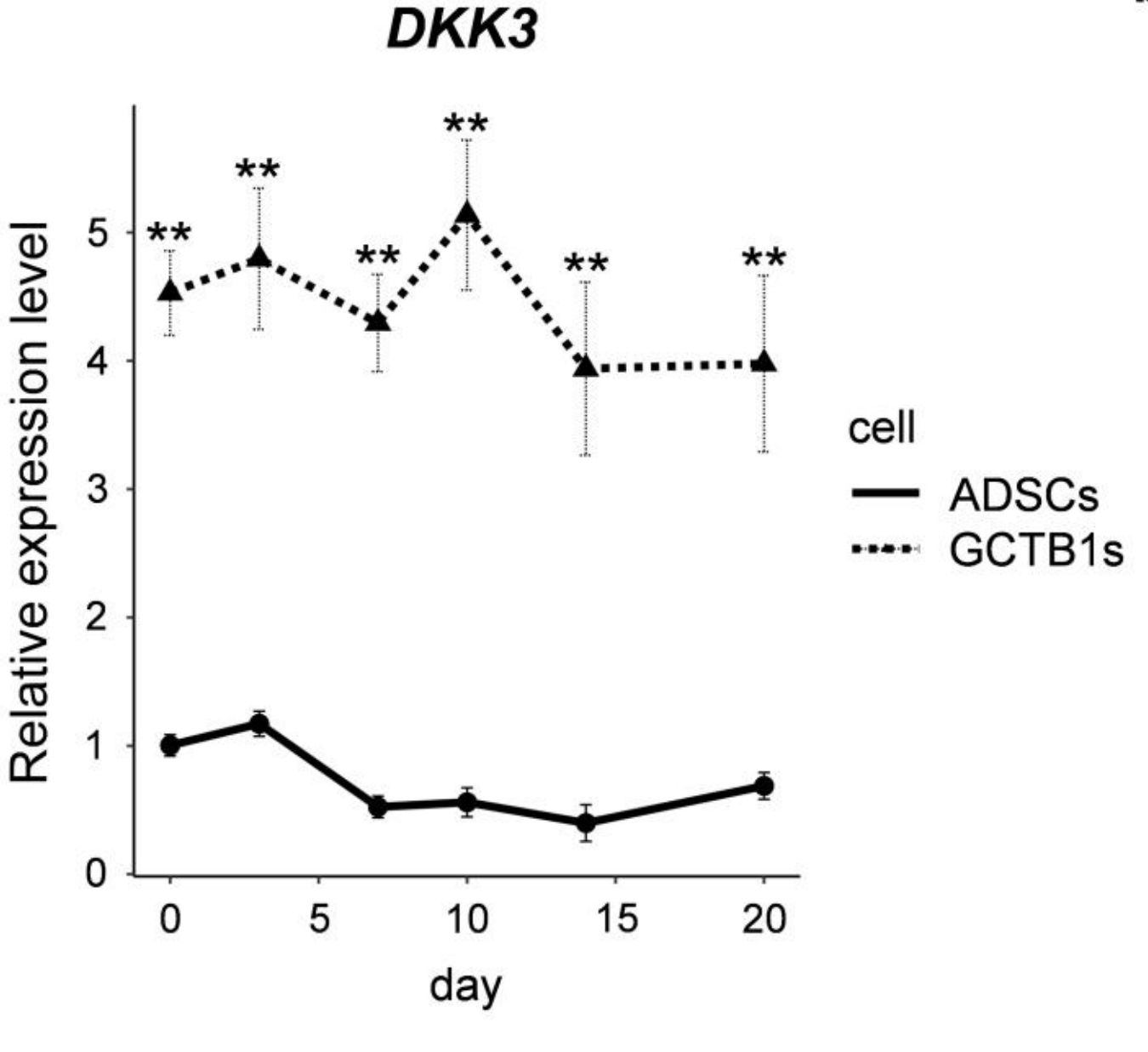

b

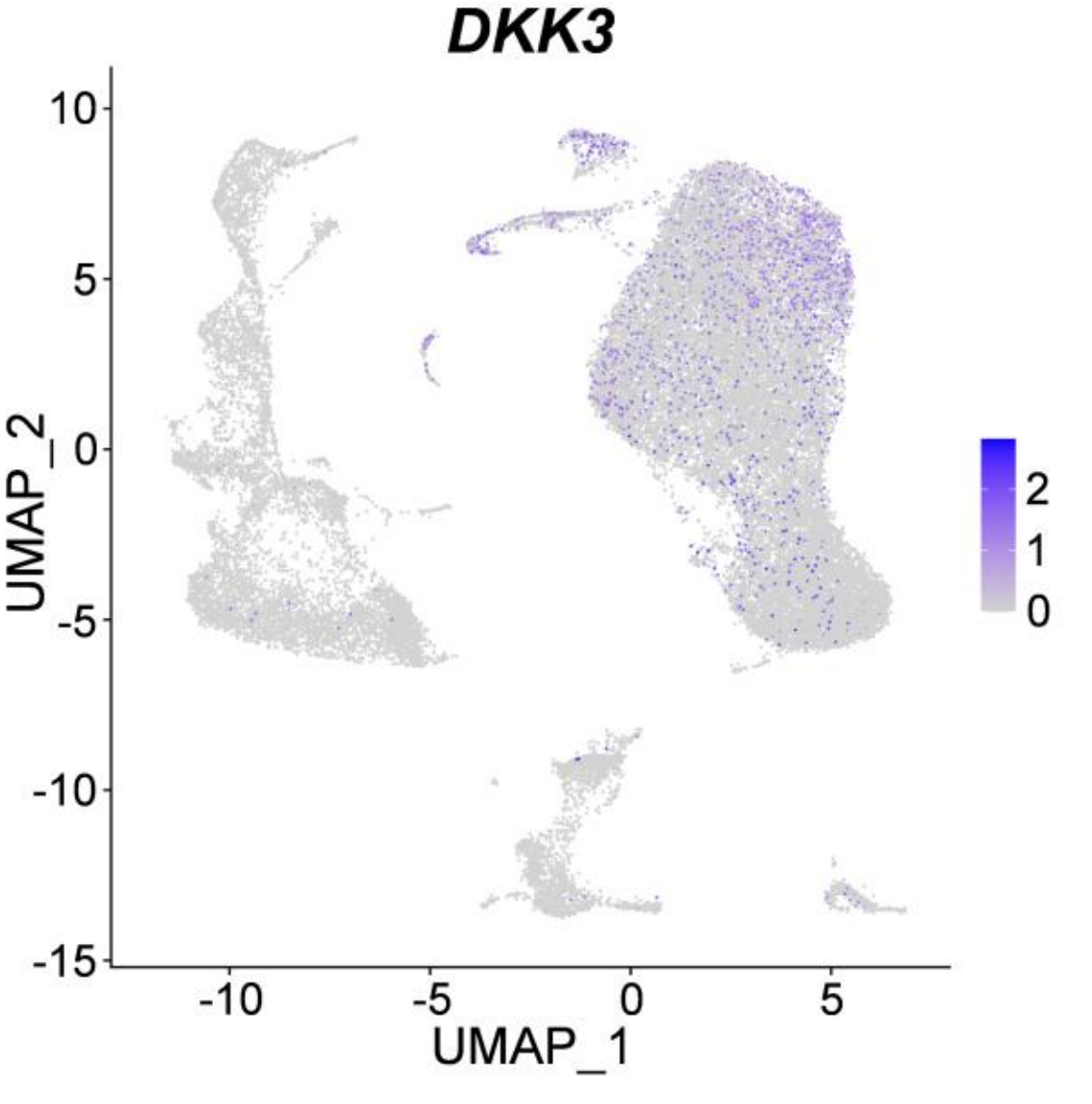

Supplement: Supplementary file 1 [file 774_2026_1713_MOESM1_ESM.pdf]
