# Supplementary material for: Giant cell tumor of bone inhibits osteoblastogenesis via WNT5B
Source: J Bone Miner Metab. 2026 Mar 23;44(4):455–68. doi: 10.1007/s00774-026-01713-3 (PMC13469380; doi:10.1007/s00774-026-01713-3)
Supplement: Supplementary file 2 [file 774_2026_1713_MOESM2_ESM.pdf]

Supplementary Information

Supplementary Figure Legend

Supplementary Fig. 1 GCTB3s express *RUNX2* and *WNT5B*

ADSCs and GCTB3s were cultured separately in osteogenic induction medium for 7, 14 or 20 days, and at each time point *RUNX2* or *WNT5B* transcript levels in these cells were analyzed by qPCR. Data represents mean indicated gene expression relative to *β-actin* (*ACTB*) ± SD in each cell (n = 6, ADSCs; n = 4, GCTB3s. Mann-Whitney U-test: *$p < 0.05$).

Supplementary Fig. 2 GCTB expresses *SFRP4* and *WNT5B*

Expression levels of genes encoding osteoblastogenesis inhibitory factors, based on analysis of GCTB scRNA-seq data.

Supplementary Fig. 3 Cells expressing *WNT5B* are distinct from those expressing *SFRP4*

(a) UMAP plot based on reanalysis of GSE147390, which contains osteoblast scRNA-seq data.

(b) Expression of osteoblast-related genes based on osteoblast scRNA-seq data.

(c) Expression of *SFRP4* and *BGLAP* (upper) or *SFRP4* and *WNT5B* (lower) based on osteoblast scRNA-seq data.

Supplementary Fig. 4 H3.3 G34W-positive GCTB stromal cells are diffusely distributed throughout the tumor tissue

The section, separated from the same paraffin block used for Visium analysis, was stained with rabbit anti-H3.3 G34W antibody followed by HRP-conjugated goat anti-rabbit Ig' antibody. The section was treated with DAB. Nuclei were stained with Hematoxylin and observed under a microscope. Bar = 50 µm.

Supplementary Fig. 5 Cells expressing *WNT5B* or *SFRP4* tend to localize to distinct regions

(a) Expression of *ACP5*, an osteoclast marker, or *CD14*, a marker of osteoclast precursors, as shown by Visium. Gray squares indicate regions of interest, which are located at the same positions as in Fig. 3c.

(b) Heatmap showing z-score—scaled average expression of *WNT5B*, *SFRP4*, *ACP5*, or *CD14* across clusters in Visium.

Supplementary Fig. 6 Cells expressing *WNT5B* or *SFRP4* are distinct from osteoclast lineage cells

UMAP plots based on GCTB scRNA-seq data showing *ACP5, CD14, WNT5B, or SFRP4* expression.

Supplementary Fig. 7 *WNT5A* expression is comparable or slightly higher in ADSCs than in GCTB1s

ADSCs and GCTB1s were cultured separately in osteogenic induction medium for 20 days, and *WNT5A* transcript levels on indicated days were analyzed by qPCR (day 20 ADSCs, A20; day 0 GCTB1s, G0; day20 GCTB1s, G20). Data represents mean *WNT5A* expression relative to *β-actin* (*ACTB*) ± SD in each cell (n = 6, A20; n = 6, G0; n = 4,

G20. Welch's test: *$p < 0.05$).

Supplementary Fig. 8 GCTB shows diffuse staining for WNT5A/B

(a) The sections, separated from the same paraffin block used for Visium analysis, were stained with rabbit anti-H3.3 G34W (left) or rabbit anti-WNT5A/B (right) antibodies followed by HRP-conjugated goat anti-rabbit Ig' antibody. Sections were then treated with DAB. Nuclei were stained with Hematoxylin and observed under a microscope. Bar = 50 μm.

(b) The sections, separated from the same paraffin block used for Visium analysis, were stained with rabbit anti-WNT5A/B antibody (left) or without primary antibody (right) followed by HRP-conjugated goat anti-rabbit Ig' antibody. Sections were then treated with DAB. Nuclei were stained with Hematoxylin and observed under a microscope. Bar = 50 μm.

Supplementary Fig. 9 Supernatants from ADSCs cultured in osteogenic induction medium increase *CTNNB1* expression in GCTB1s

ADSCs were cultured in osteogenic induction medium and supernatants were collected on days 7 (Sup.day7), 21 (Sup.day21) or 24 (Sup.day24). GCTB1s were then cultured in each of the collected ADSC supernatants. Control GCTB1s were cultured in osteogenic induction medium only. After 6 hours of culture, mRNA was collected and qPCR was performed to analyze *CTNNB1* expression. Data represents mean *CTNNB1* expression relative to *β-actin* (*ACTB*) ± SD in each culture ($n = 6$ each, Welch's test: *$p < 0.05$).

Supplementary Fig. 10 Proposed survival strategy of GCTB cells

GCTB cells inhibit the differentiation of surrounding osteoblast-lineage cells via paracrine WNT5B. GCTB cells may originate from *WNT5B*-positive cells.

Supplementary Fig. 11 *WNT5B* expression in GCTB1s is significantly increased by supernatants from osteogenic induction cultures of ADSCs at the specific time point

GCTB1s were cultured in osteogenic induction medium (as control) or the culture supernatant from late stage (day 21) of ADSC osteoblastic differentiation (Sup.day21). After indicated time points, mRNA was collected and qPCR was performed to analyze *WNT5B* expression. Data represents mean *WNT5B* expression relative to *β-actin* (*ACTB*) ± SD in each culture (n = 5 each, 1-hour; n = 6 each, 6-hour; n = 6 each, 48-hour; n = 7 (control) and n = 5 (Sup.day21), 4-day, Mann-Whitney U-test: **$p < 0.01$).

Supplementary Fig. 12 *WNT5B* knockout GCTB1s (G1KO) failed to induce mineralization

G1KO were cultured in osteogenic induction medium and stained with Alizarin Red S on day 20. Bar = 200 µm.

Supplementary Fig. 13 GCTB1s express *DKK3*

(a) ADSCs and GCTB1s were cultured separately in osteogenic induction medium for 3, 7, 10, 14 or 20 days, and at each time point *DKK3* transcript levels in these cells were analyzed by qPCR. Data represents mean indicated gene expression relative to *β-actin* (*ACTB*) ± SD in each cell (n = 6 each, Mann-Whitney U-test: **$p < 0.01$).

(b) UMAP plots based on GCTB scRNA-seq data showing *DKK3* expression.

Supplementary method

Processing of scRNA-seq data

To investigate the characteristics of OBs, raw data were downloaded from GSE147390 (Liu et al. 2021) in the Gene Expression Omnibus (GEO) database. Raw FASTQ file of ALP+/CD45/34/31- OBs (GSM4429895) was reanalyzed. Raw reads were aligned to the GRCh38 reference genome, unique molecular identifier (UMI) counting was performed using Cell Ranger Package (version 7.0.1) pipeline with default parameters. Seurat package (version 5.0.1) was used for quality filtering and subsequent analysis. Low-quality cells (< 200 genes/cell, > 5000 genes/cell and 20% mitochondrial genes) were excluded. The clusters expressing *COL1A1* were identified. Following reclustering, 8063 cells were retained. Principal component analysis (PCA) with "RunPCA" function was performed to reduce the dimensionality of scRNA-seq data. To identify clusters, the top 20 principal components were selected and subjected to "FindNeighbors", "FindClusters", and "RunUMAP" (Uniform Manifold Approximation and Projection [UMAP]) functions. The resolution parameter was set to 0.5 unless otherwise specified. The cluster-specific marker genes were identified using the Wilcoxon test implemented in "FindAllMarkers" function. The cell clusters were manually annotated according to these marker genes.
